# Single-cell RNA sequencing analysis identifies acute changes in the tumor microenvironment induced by interferon α gene therapy in a murine bladder cancer model

**DOI:** 10.3389/fimmu.2024.1387229

**Published:** 2024-11-04

**Authors:** Alexis R. Steinmetz, Morgan Pierce, Alberto Martini, Come Tholomier, Ganiraju Manyam, Yan Chen, Akshay Sood, Jonathan J. Duplisea, Burles A. Johnson, Bogdan A. Czerniak, Byron H. Lee, Chinnaswamy Jagannath, Seppo Yla-Herttuala, Nigel R. Parker, David J. McConkey, Colin P. Dinney, Sharada Mokkapati

**Affiliations:** ^1^ Department of Urology, The University of Texas MD Anderson Cancer Center, Houston, TX, United States; ^2^ Department of Bioinformatics and Computational Biology, The University of Texas MD Anderson Cancer Center, Houston, TX, United States; ^3^ Johns Hopkins Greenberg Bladder Cancer Institute, Brady Urological Institute, Johns Hopkins University, Baltimore, MD, United States; ^4^ Department of Pathology, The University of Texas MD Anderson Cancer Center, Houston, TX, United States; ^5^ Department of Pathology and Genomic Medicine, Houston Methodist Research Institute, Houston, TX, United States; ^6^ A.I. Virtanen Institute for Molecular Sciences, Kuopio, Finland

**Keywords:** nadofaragene, gene therapy, interferon α, bladder cancer, single-cell RNA sequencing, cytokine

## Abstract

**Introduction:**

Nadofaragene firadenovec (Ad-IFNα/Syn3) is now approved for BCG-unresponsive bladder cancer (BLCA). IFNα is a pleiotropic cytokine that causes direct tumor cell killing via TRAIL-mediated apoptosis, angiogenesis inhibition, and activation of the innate and adaptive immune system. We established an immunocompetent murine BLCA model to study the effects of murine adenoviral IFNα (muAd-Ifnα) gene therapy on cancer cells and the tumor microenvironment using a novel murine equivalent of Nadofaragene firadenovec (muAd-Ifnα).

**Methods:**

Tumors were induced by instilling MB49 cells into the bladders of mice; luciferase imaging confirmed tumor development. Mice were treated with adenovirus control (Ad-Ctrl; empty vector), or muAd-Ifnα (3x10^11^ VP/mL), and survival analysis was performed. For single-cell sequencing (scRNAseq) analysis (72h), bladders were harvested and treated with collagenase/hyaluronidase and TrypLE for cell dissociation. Single cells were suspended in PBS/1% FBS buffer; viability was assessed with Vicell cell counter. scRNAseq analysis was performed using 10X genomics 3’ sequencing. Raw RNAseq data were pre-processed using Cell Ranger single-cell software. Seurat (R package) was used to normalize and cluster the scRNA data. Pooled differential gene expression analysis in specific cell clusters was performed with DESeq2.

**Results:**

We identified 16 cell clusters based on marker expression which were grouped into epithelial (tumor), uroplakin-enriched, endothelial, T-cells, neutrophils, and macrophage clusters. Top differentially expressed genes between muAd-Ifnα and Ad-Ctrl were identified. Within the specific cell clusters, IPA analysis revealed significant differences between muAd-Ifnα and control. IFNα signaling and hypercytokinemia/chemokinemia were upregulated in all clusters. Cell death pathways were upregulated in tumor and endothelial clusters. T-cells demonstrated upregulation of the immunogenic cell death signaling pathway and a decrease in the Th2 pathway genes. Macrophages showed upregulation of PD1/PD-L1 pathways along with downregulation of macrophage activation pathways (alternate and classical). Multiplex immunofluorescence confirmed increased infiltration with macrophages in muAd-Ifnα treated tumors compared to controls. PD1/PD-L1 expression was reduced at 72h.

**Discussion:**

This single-cell analysis builds upon our understanding of the impact of Ad-IFNα on tumor cells and other compartments of the microenvironment. These data will help identify mechanisms to improve patient selection and therapeutic efficacy of Nadofaragene firadenovec.

## 1 Introduction

A significant portion of patients with non-muscle-invasive bladder cancer (NMIBC) treated with frontline intravesical *Bacillus Calmette-Guérin* (B*CG*) will not achieve long-term recurrence and progression-free survival (1). The definitive treatment for these patients is extirpative surgery which is associated with significant morbidity and mortality (1). In December 2022, intravesical interferonα (IFNα) gene therapy with Nadofaragene firadenovec (Ad-IFNα/Syn3) was approved for patients with BCG-unresponsive NMIBC (2).

Ad-IFNα/Syn3 is a non-replicating adenoviral vector that delivers a copy of the human IFNα2b transgene to bladder cancer and normal bladder urothelium, leading to the production of IFNα protein (3). Well-established cytotoxic, anti-angiogenic, and immunogenic antitumor properties of this pleiotropic cytokine underly the efficacy of Nadofaragene firadenovec (Nadofaragene) in bladder cancer (BLCA) (4–8). In the phase III trial, treatment with intravesical Nadofaragene provided an impressive durable 12-month response: 60% for high-grade Ta/T1 disease and 46% for CIS in those who had a complete response at 3 months (9). Despite these encouraging findings, additional studies are warranted to improve patient selection and therapeutic efficacy.

Given the complex immunostimulatory and immunosuppressive effects of IFNα, understanding its impact on the tumor microenvironment (TME) in BLCA is of vital significance. Rapid advancements in single-cell RNA sequencing (scRNAseq) have provided insights into the cellular heterogeneity of tumors and the TME that were not appreciated through bulk RNA sequencing (10). Using known cluster-specific genes, scRNAseq has been used to identify the different bladder tumor-infiltrating cell types and allows for analysis of differentially expressed genes among individual cells (11, 12).

Prior preclinical experiments with Ad-IFNα took place in orthotopic nude mice models using human Ad-IFNα, which did not allow for an analysis of the systemic immune response or TME (7). In this context, we developed an immunocompetent murine BLCA model to study the effects of IFNα on cancer cells and the TME using a novel murine equivalent of Nadofaragene (muAd-Ifnα). We confirmed effective transgene expression by murine BLCA cells and performed scRNAseq and functional enrichment analysis on the cell clusters identified from mice treated with muAd-Ifnα. The objective of the present study is to define acute changes in the tumor microenvironment induced by muAd-Ifnα at 72h post treatment, at which time transduction of tumor and subsequent expression of RNA and proteins occurs. This novel data demonstrates the transcriptional impact of muAd-Ifnα on tumor cells and other cellular compartments of the TME and identifies distinct mechanisms unique to these cell types. We also correlated PD-1 and PD-L1 expression following acute exposure to muAd-Ifnα using multiplex immunofluorescence.

## 2 Materials and methods

### 2.1 Cell culture

Mouse urothelial cell line MB49/GFP-luciferase was a generous gift from Dr. Robert Svatek (The University of Texas Health, San Antonio). Cells were cultured in minimum essential media (supplemented with 10% fetal bovine serum and 1% penicillin/streptomycin. For viral transduction, 50,000 cells were seeded in 6-well culture dishes. After overnight attachment, media was replaced with polybrene-containing media (4 μg/mL), and viral particles were added at a multiplicity of infection (MOI) 100 or 500. Recombinant murine interferon 100 U/mL (muIFNα) was added as the control. Endothelial cells 2H-11 were cultured in DMEM and a tube formation assay was performed as previously described (13).

### 2.2 Viral vectors

Adenoviral vectors for murine IFNα11 were developed for this study using Invitrogen’s Gateway cloning technology. FKD Therapies (University of Finland) provided murine adenoviral vectors (Ad-Ctrl and muAd-Ifnα). Briefly, Invitrogen’s Gateway cloning technology was used for cloning murine IFNα11 gene into pDONR211 vector to obtain pENTRY muIFNα11 and then subcloned into shuttle vector pAdApt-muIFNa11 to generate adenoviral muIFNα11 vectors. This vector is transfected into 293T cells and western blotting was performed from cell lysates prepared from these cells to detect 21 kDa mIFNα11 protein (**Supplementary Figure 1A**). The titer of the virus is provided by the company and an MOI of 100 equals 100 viral particles per cell.

### 2.3 Q-PCR analysis

RNA was extracted using an Ambion miRVANA kit (AM1561) and quantified using NanoDrop ND-1000 spectrophotometer. 20 ng of RNA was used along with AgPAth-ID One-Step RT-PCR kit (Thermo Fisher, 4387391) with Taqman probes to detect Irf7 (Mm00516788_m1), CD274 (Mm00452054), Tnfsf10 (Mm01283606_m1) and GAPDH (Mm99999915_g1) on a StepOnePlus Real-Time PCR instrument. The comparative Ct method was used to determine relative gene expression.

### 2.4 ELISA for murine IFNα

Cell-free supernatants from cells treated with the adenoviral vectors were collected and levels of muIFNα were measured by ELISA (PBL, 42115-1).

### 2.5 Western blotting

**Figure 1:**
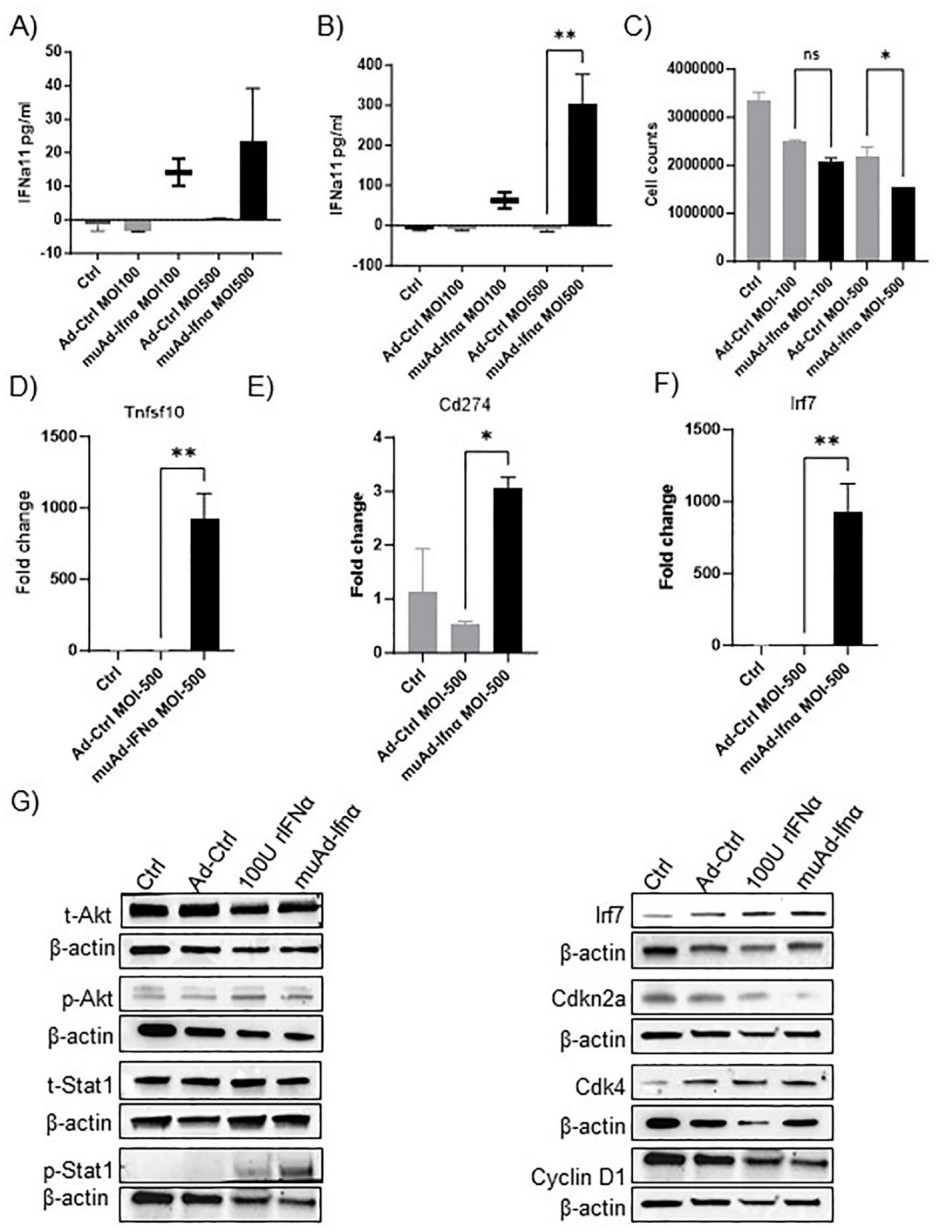
muAd-Ifnα as a therapeutic agent for treating murine BLCA models. ELISA for muIFNα in cell-free supernatants in muAd-Ifnα treated cells (MOI 100 and MOI 500) compared to Ad-Ctrl MB49 cells at 24h post-transduction **(A)** and 72h after post-transduction **(B)**. Cell counts at 72h showed decreased numbers at MOI 500 in muAd-Ifnα treated cells **(C)**. Q-PCR analysis showing increased expression of Trail (Tnfsf10) **(D)**, Pd-l1 (Cd274) **(E)**, and Irf7 **(F)**. Western blot analysis showing upregulation of IFNα signaling molecules including p-Akt, p-Stat1, Irf7; downregulation of cell cycle regulator Cdkn2a and Cyclin D1 and upregulation of Cdk4 in cells treated with recombinant IFNα (rIFNα) or muAd-Ifnα when compared to Ctrl and Ad-Ctrl **(G)**. * p < 0.05; ** p < 0.01; ns-not significant.

Cells were grown in monolayers and treated with adenoviral vectors. After 48 hours, cells were washed once with phosphate-buffered saline (PBS) and scraped off the plates into whole cell lysis buffer (50 mM Tris-HCl, pH 7.4; 150 mM NaCl; 5 mM EDTA; 25 mM NaF; 1% Triton-X 100; 1% NP-40; 0.1 mM Na3VO4; 12.5 mM *β*-glycerophosphate; 1 mM PMSF) containing protease and phosphatase inhibitors. Cell lysates were prepared by incubation on ice for 30–40 minutes with intermittent vortexing every 10 minutes. Protein concentration was measured (Micro BCA protein assay kit, Thermo Fisher), and 30 μg of protein was resolved on 4-15% gradient gel (Bio-Rad) and transferred to a nitrocellulose membrane. The blots were blocked in 10% milk powder in PBS and probed with antibodies against PD-L1 (AF1019, Bio-Techne), STAT1 (33-1400, Invitrogen), pSTAT1 (Cell Signaling, #9171) and β-actin (AC-74). Species-specific secondary antibodies were used to detect protein bands using an enhanced chemiluminescence detection kit (GE Healthcare). Cells were treated in triplicates; we performed the experiments at least twice and representative western blots from one experiment are provided in **Figure 1**.

### 2.6 *In vivo* experiments

**Figure 2:**
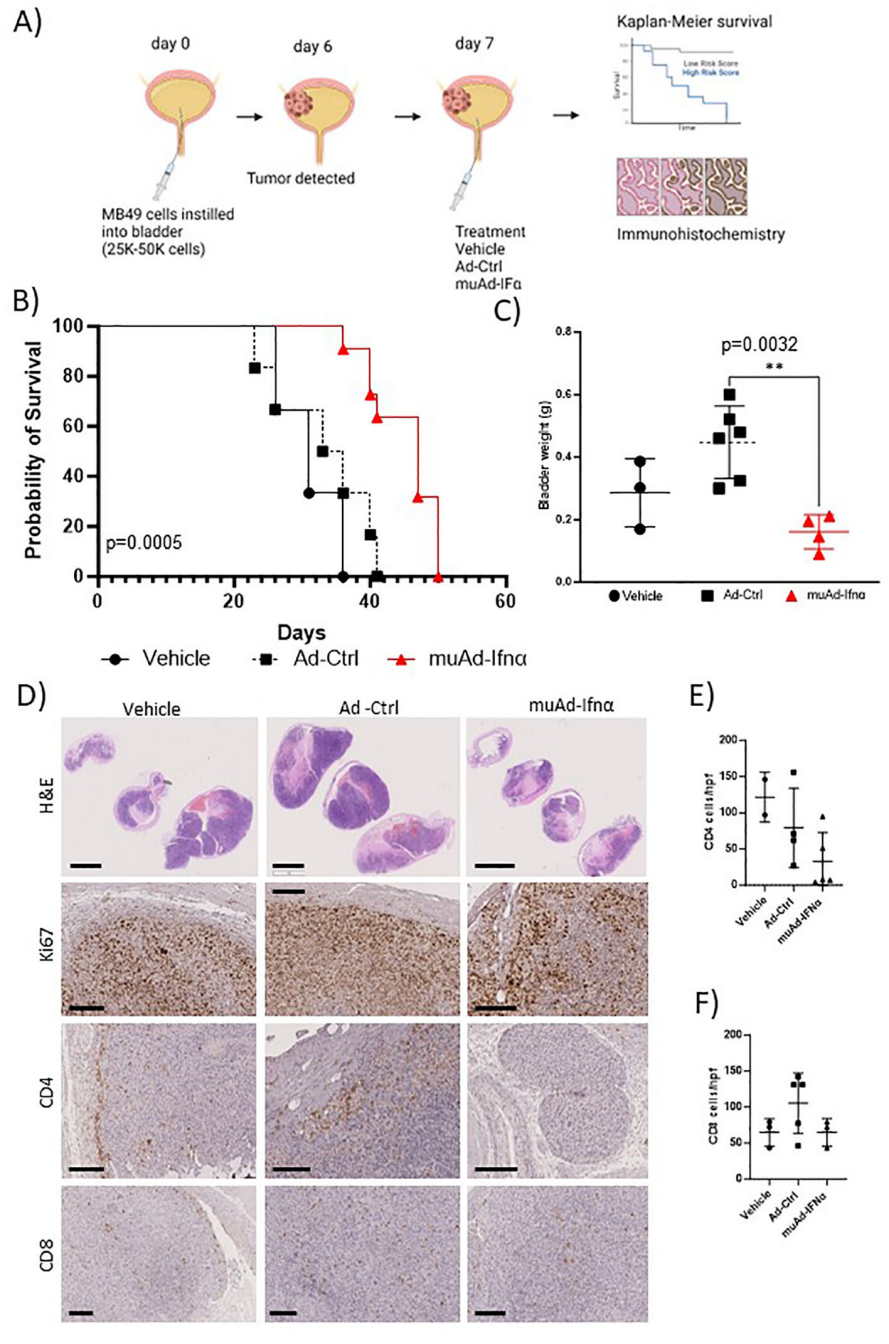
Preclinical assessment of muAd-Ifnα in an intravesical MB49 BLCA model. Schematic representation of preclinical study **(A)**. Kaplan-Meier survival plot showing a significant difference in percent survival in the C57Bl/6 MB49 intravesical model treated with Ad-Ctrl and muAd-Ifnα (p=0.0005; **B**). Bladder weight showing a significant difference between Ad-Ctrl and muAd-Ifnα treated mice (p=0.0032; **C**). Histology of bladders showing decreased tumor burden in muAd-Ifnα treated mice when compared to no treatment or Ad-Ctrl mice (**D**, top panel); decreased proliferation as shown by Ki67 staining (**D**, second panel); Cd4 immunohistochemistry (**D**, third panel) and Cd8 immunohistochemistry (**D**, bottom panel). Quantification of immune cells per HPF showing decreased Cd4 expression in muAd-Ifnα treated tumors **(E)** and similar cell numbers for CD8 cells **(F)**. ** p < 0.01.

All animal experiments were conducted in compliance with the Institutional Animal Care and Use Committee guidelines at The University of Texas MD Anderson Cancer Center (Houston, TX). MB49 bladder tumors were established following intravesical instillation using previously published protocols (14). Briefly after anesthetizing mice with isoflurane, the mouse’s urethra was catheterized and instilled with 100 μL of PLL (0.01 μg/mL) for 10 minutes. After emptying the bladder, 50,000 MB49 cells in 100 μL of HBSS were instilled into mouse bladders for 30 minutes. Mice were allowed to recover, and tumor burden was assessed by IVIS Spectrum *In vivo* Imaging System and Living Image Software (Perkin Elmer). Mice were treated intravesically with either Syn3 (1 mg/ml, vehicle), Ad-Ctrl (Control adenoviral vector without IFNα11 gene; 3X10^11^ virus particles) or muAd-Ifnα (adenoviral vector with murine Ifna11 gene; 3X10^11^ virus particles) and tumor growth was monitored twice weekly. Mice with a significant reduction in body weight, lethargy, and hematuria were deemed moribund and were humanely euthanized. All experiments were repeated thrice, and one representative survival experiment is shown in **Figure 2**.

**Figure 3:**
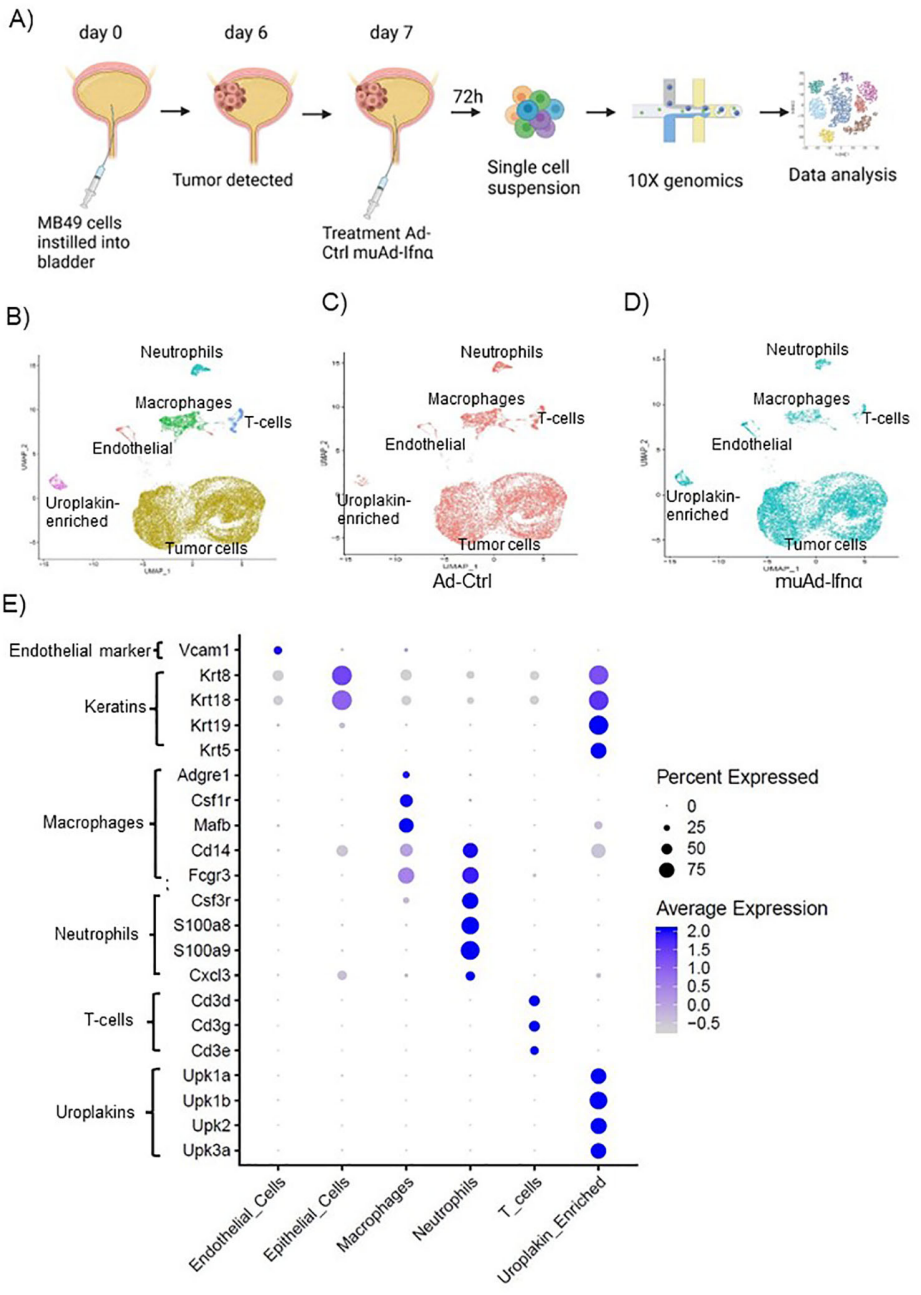
High-throughput 10X scRNAseq analysis of murine bladders treated with muAd-Ifnα. Schematic representation of scRNAseq in an intravesical BLCA model using 10X platform **(A)**. UMAP visualization representing all cells of mice treated with Ad-Ctrl and muAd-Ifnα showing distinct cell clusters differentially colored and labeled: tumor cells - yellow, endothelial cells - orange, T-cells - blue, macrophages - green, Uroplakin-enriched - pink, neutrophils - blue-green **(B)**. UMAP visualization showing the distribution of cells from treatment with Ad-Ctrl (pink dots) and muAd-Ifnα (blue dots) in all the cell clusters **(C, D)**. Dot plot showing expression of marker genes differentially expressed across the 6 cell clusters identified by UMAP **(E)**.

### 2.7 scRNAseq analysis

For single cell analysis, female C57Bl/6 mice were instilled with MB49 cells and treated with Ad-Ctrl or muAd-Ifnα one week after tumor cell instillation. 72 hours after treatment, bladders were collected and washed in sterile DPBS in a 100 mm dish with 0.5 mL of media supplemented with 5% FBS. The bladder was dissected into small 1-2 mm pieces and transferred to a 15 mL tube with 3.5 mL media. 0.2 mL of collagenase/hyaluronidase mixture was added to the media and incubated at 37 degrees Celsius in an orbital shaker for 3 hours at 200 rpm. After tissue digestion, an equal volume of media was added to the tube to stop digestion. Dissociated cells were strained through a 100 μm cell strainer and cells were centrifuged and washed twice with DPBS and resuspended in 1.5 mL DPBS. 0.1 mL of a cell suspension was examined for viability using the Vi-cell cell viability analyzer. 10x genomics was performed on the cells isolated using 3’ RNA sequencing. This experiment was performed once and 2 animals per group were used to generate the data.

### 2.8 scRNAseq data analysis

The raw single-cell RNAseq data were aligned to the mouse genome (mm10) and pre-processed using the Cell Ranger pipeline from 10x Genomics. Cells containing less than 100 gene features, or more than 20% mitochondrial gene counts were filtered out to minimize low quality cells (15, 16). R package Seurat was utilized to normalize unique molecular identifier (UMI) counts and cluster the RNA expression data by log-normalization and Louvain algorithm respectively (17). Expression clusters with extremely low gene features were further filtered out for downstream analysis. Cells were classified into specific types based on pre-defined marker gene expression in the clustered data. Differential Expression analysis was performed between treatment groups on individual cell types using cells expressing a minimum of 500 genes using R package DESeq2 after pooling counts across the samples (18). Significantly differentially expressed genes were defined using an FDR cutoff of 0.05 and log2 fold change of 1. Further downstream pathway exploration was performed by pre-ranked gene set enrichment analysis based on log2 fold change among treatment groups across the cell types using the Hallmark and KEGG pathway databases (19). The pathways and targets were generated through the use of QIAGEN Ingenuity Target Explorer (20).

### 2.9 Histology and immunostaining

Mouse tissues were fixed in buffered formalin, embedded in paraffin, and sectioned at the Research Histology core laboratory at MD Anderson Cancer Center. Immunostaining was performed with the specified antibodies, and species-specific horseradish peroxidase-conjugated secondary antibodies were used to detect proteins using the 3,3-diaminobenzidine substrate kit (Vector Laboratories). Sections were counterstained with hematoxylin and mounted in Permount. Images were captured using a Nanozoomer image scanner (Hamamatsu). For multiplex immunofluorescence, we used a mouse panel optimized by the Department of Translational Molecular Pathology at MD Anderson. We included CD3ϵ, CD4, CD8, PD-1, PD-L1, F4/80, CK19 proteins, and DAPI was used to stain the nuclei. All multiplex immunofluorescence samples were blinded and analyzed by a pathologist.

### 2.10 Statistical analysis

**Figure 4:**
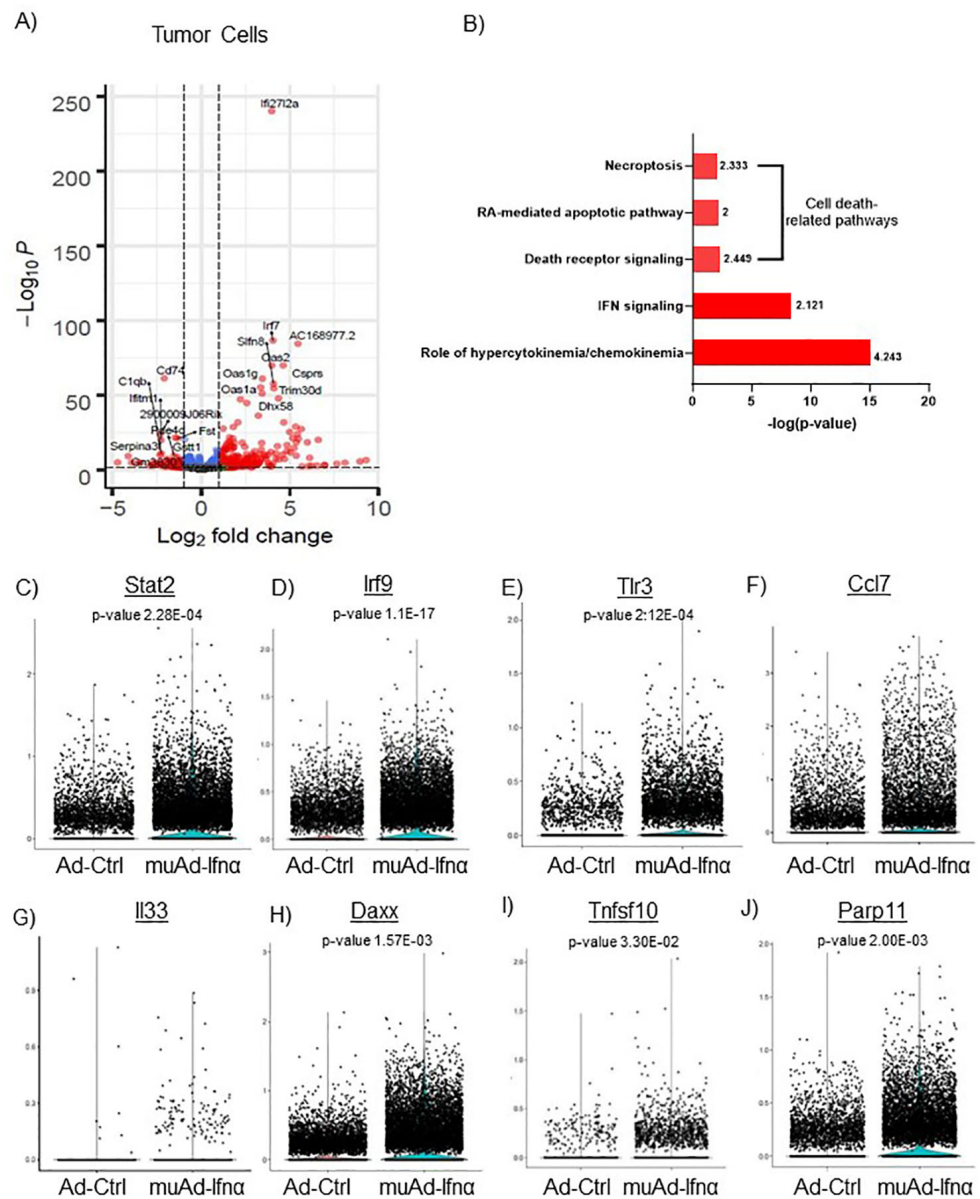
scRNAseq analysis comparing Ad-Ctrl and muAd-Ifnα in the tumor cells. Volcano plot showing differentially expressed genes (DEGs) up and downregulated in tumor cells (FDR 0.05; log2 fold change (FC) of 1 **(A)**. Ingenuity pathway analysis (IPA) showing differentially expressed pathways treated with muAd-Ifnα compared with Ad-Ctrl showing positive pathway enrichment for IFNα signaling and hypercytokinemia/chemokinemia, as well as cell death pathways including necroptosis, RA-mediated apoptotic pathway, and death receptor pathway **(B)**; z-score for enrichment is indicated above each bar. Comparison of IFNα target genes (Stat2, Irf7, Tlr3), cytokines (Ccl7 and IL-33), and apoptosis pathway genes (Daxx, Tnfsf10, Parp11) at single cell level between Ad-Ctrl and muAd-Ifnα treated cells. p-values are indicated **(C–J)**.

**Figure 5:**
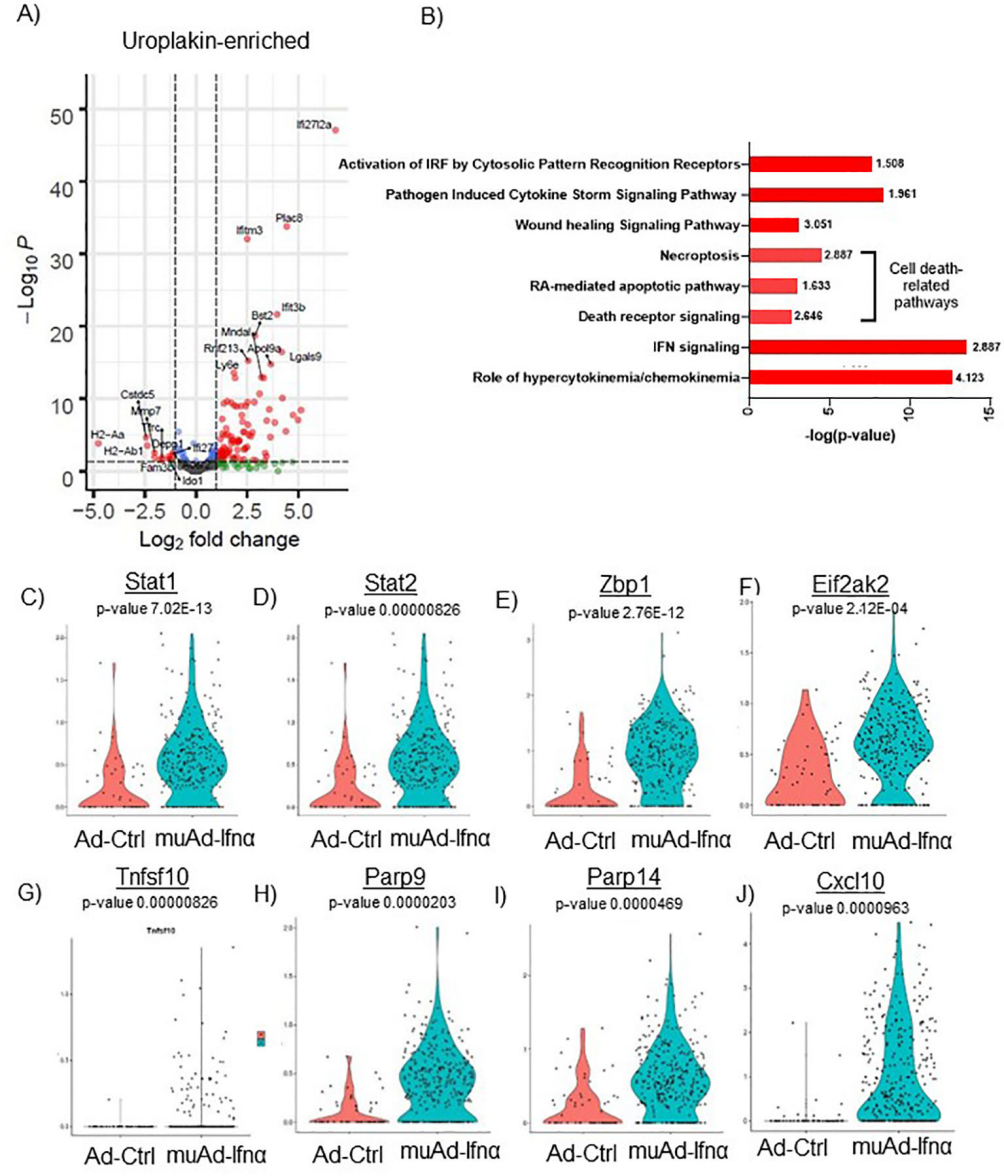
scRNAseq analysis comparing Ad-Ctrl and muAd-Ifnα in uroplakin-enriched cells. Volcano plot showing DEGs up and downregulated in the uroplakin-enriched cells (FDR 0.05; log2 FC 1) **(A)**. IPA showing differentially regulated pathways in uroplakin-enriched cells treated with muAd-Ifnα **(B)**. z-score is indicated above each bar. Comparison of IFNα target genes (Stat1, Stat2, Zbp1, Eif2ak2), cell death pathway genes (Tnfsf10, Parp9, Parp14), chemokines (Cxcl10) at single cell level between Ad-Ctrl and muAd-Ifnα treated cells **(C–J)**.

**Figure 6:**
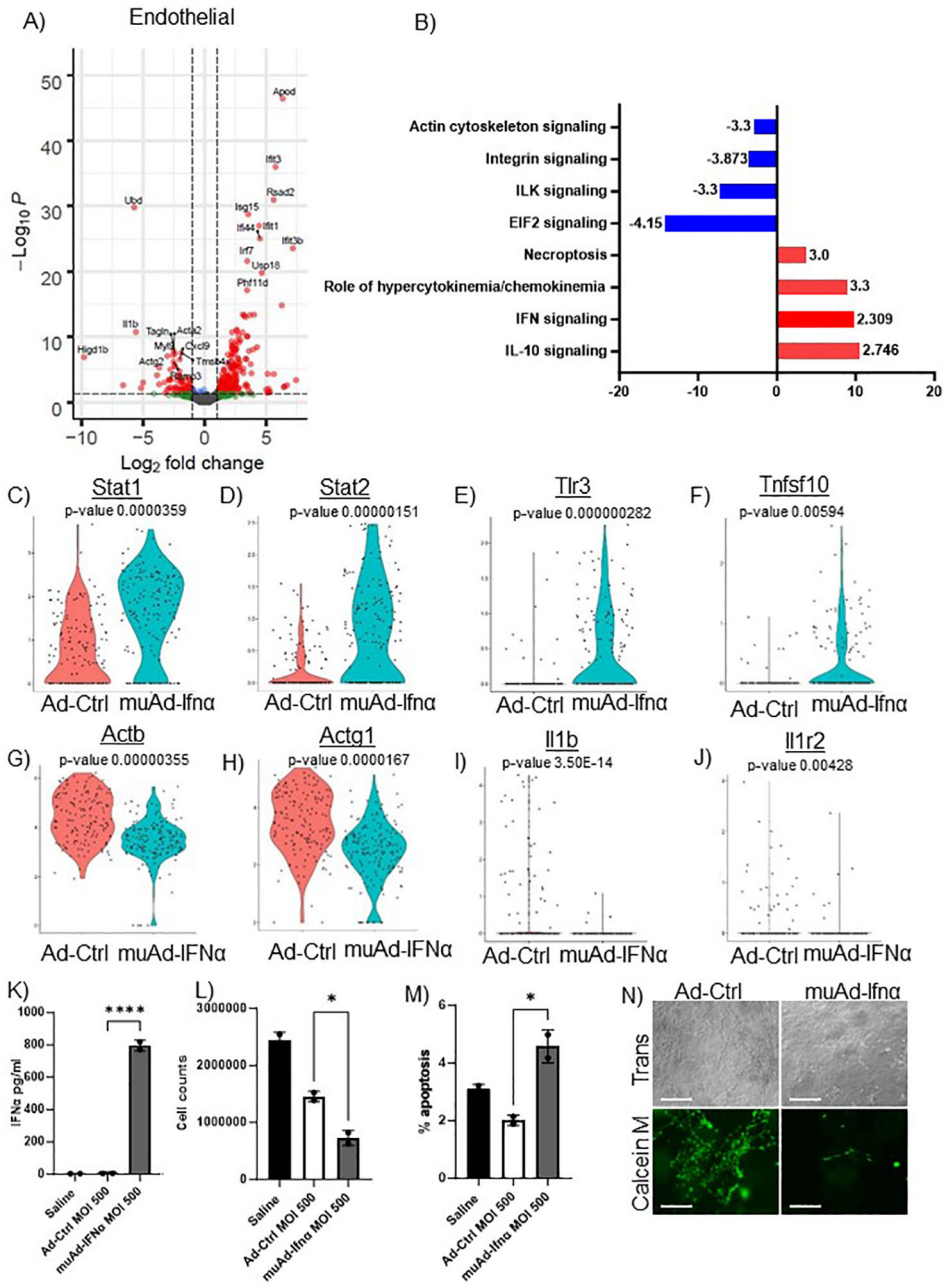
scRNAseq analysis comparing Ad-Ctrl and muAd-Ifnα in the endothelial cells. Volcano plot showing DEGs up and downregulated in the endothelial cells (FDR 0.05; log2 FC 1) **(A)**. IPA showing differentially regulated pathways in endothelial cells treated with muAd-Ifnα **(B)**. z-score is indicated above each bar. Comparison of IFNα target genes (Stat1, Stat2, Tlr3), cell death pathway genes (Tnfsf10), actin cytoskeletal genes (Actb, Actg1) and chemokine IL-1β and IL-1r2 at single cell level between Ad-Ctrl and muAd-Ifnα treated cells (p-values are indicated) **(C–J)**. IFNα ELISA **(K)**, cell counts **(L)**, and percent apoptotic cells **(M)** in murine endothelial cell line 2H-11 treated with Ad-Ctrl and muAd-Ifnα. Tube formation assay of 2H-11 cells treated with Ad-Ctrl and muAd-Ifnα treated cells showing transmission microscopic images (**N**, top panel) and calcein M-stained cells (**N**, bottom panel). * p < 0.05; **** p < 0.0001.

**Figure 7:**
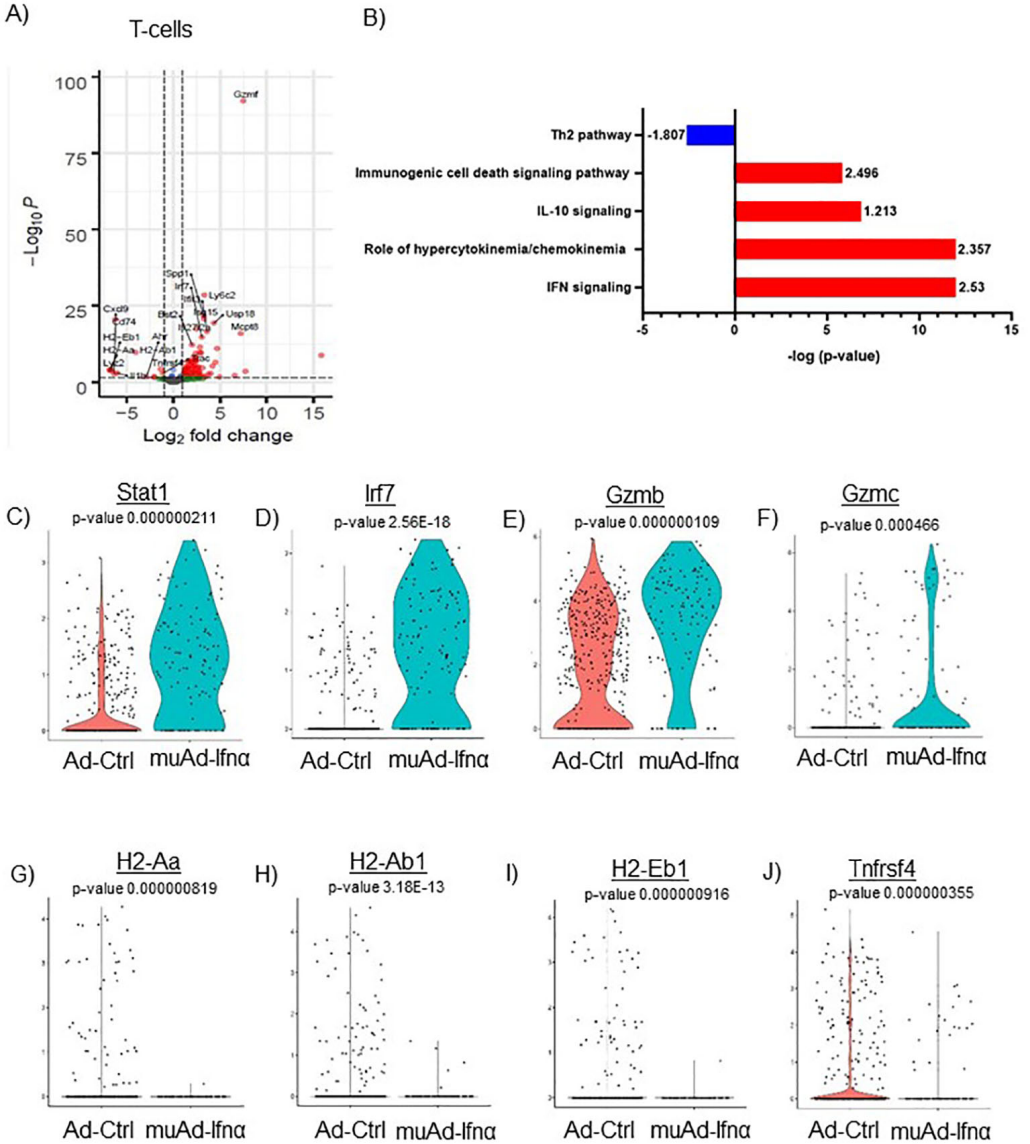
scRNAseq analysis comparing Ad-Ctrl and muAd-Ifnα in the tumor-infiltrating T cells. Volcano plot showing DEGs up and downregulated in the tumor-infiltrating T cells (FDR 0.05; log2 FC 1) **(A)**. IPA showing differentially regulated pathways in T-cells treated with muAd-Ifnα **(B)**. z-score is indicated above each bar. Comparison of IFNα target genes (Stat1, Irf7), cytotoxic T cell markers (Gzmb, Gzmc) and Th2 markers (H2-Aa, H2-Ab1, H2-Eb1 and Tnfrsf4 at single cell level comparing Ad-Ctrl and muAd-Ifnα treated T cells. p-values are indicated. **(C–J)**.

**Figure 8:**
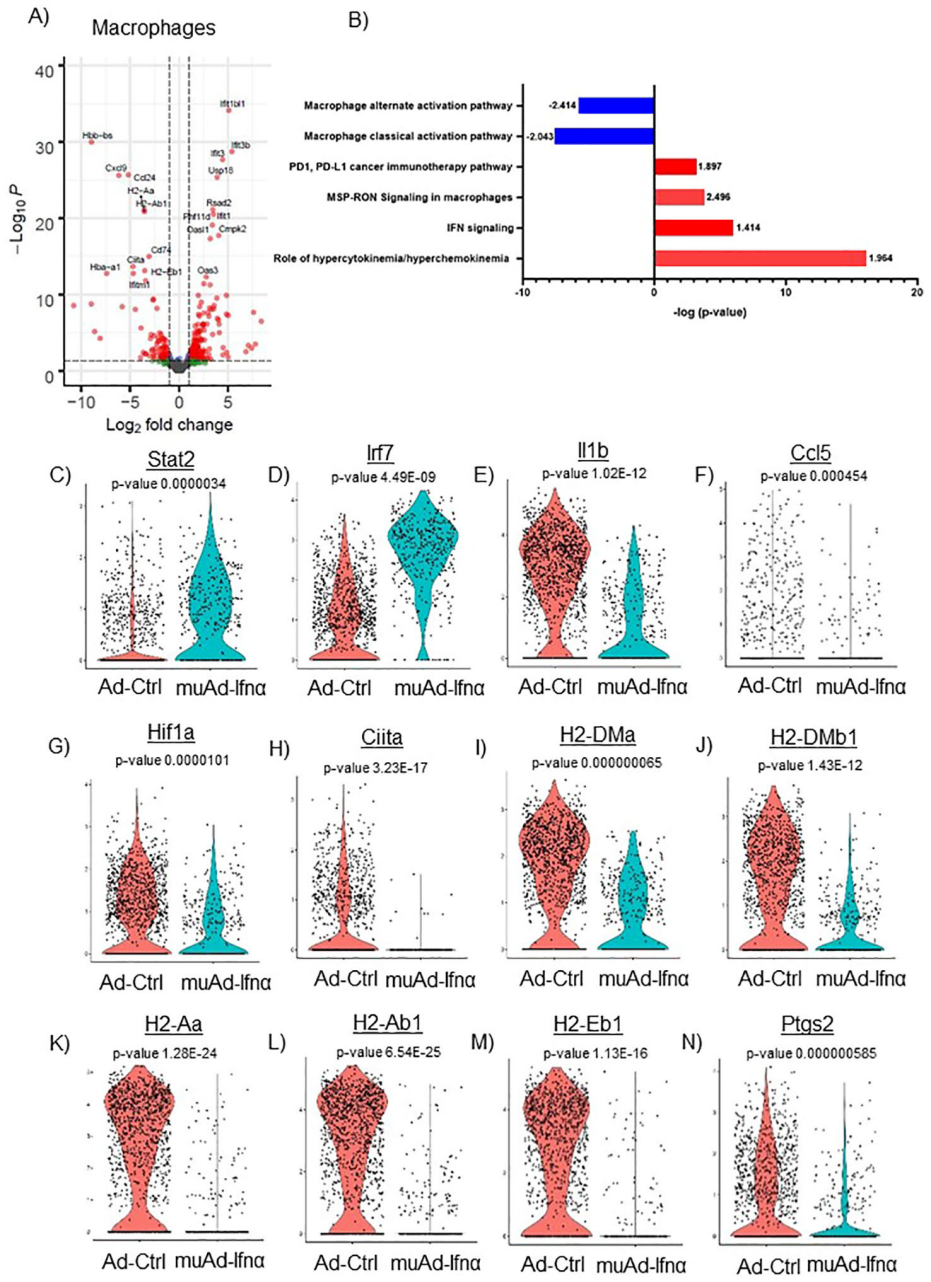
scRNAseq analysis comparing Ad-Ctrl and muAd-Ifnα in the macrophages. Volcano plot showing DEGs up and downregulated in macrophages (FDR 0.05; log2 FC 1) **(A)**. IPA showing differentially regulated pathways in macrophages treated with muAd-Ifnα **(B)**. z-score is indicated above each bar. Comparison of IFNα target genes (Stat1, Irf7), cytokines (IL-1β, Ccl5), and macrophage classical activation pathway markers (Hif1α, Ciita, H2-Dma, H2-Dmb1, H2-Aa, A2-Ab1, H2-Eb1, and Ptgs2) at the single cell level comparing Ad-Ctrl and muAd-Ifnα treated macrophages. p-values are indicated **(C–N)**.

Statistical analysis was performed using GraphPad Prism 7 software. ANOVA was used to make multiple group comparisons and results were considered significant when the p-value was < 0.05 (**Figures 1**, **2**). A log-rank test was used to perform the survival analysis (**Figure 2**). p-values in violin plots (**Figures 4**–**8**) are generated from differential expression analysis, and Wald tests were performed using DESeq2 package of R to compare groups.

## 3 Results

### 3.1 Developing muAd-Ifnα as a therapeutic agent for treating murine BLCA

We developed an adenoviral vector expressing muIFNα11 and tested its anti-tumor efficacy in murine cell lines and animal models. The syngeneic murine BLCA cell line MB49 was treated *in-vitro* at multiplicity of infection (MOI) of 100 and 500 with Ad-Ctrl or muAd-Ifnα vectors, and cell-free supernatants were collected 24h and 72h after transduction (**Figures 1A, B**). muIFNα11-specific ELISA confirmed robust and significant production of muIFNα protein (305.3 pg/ml at MOI 500) when compared to undetectable levels in Ctrl (untreated) or Ad-Ctrl (treated) cells at 72h post-transduction (**Figure 1B**). A significant reduction in cell numbers was also noted at 72h post-transduction with muAd-Ifnα at an MOI 500 (**Figure 1C**). Quantitative real-time PCR confirmed induction of IFNα target genes Tnfsf10 (919-fold), Cd274 (3-fold), and Irf7 (932.5-fold) 24h after transduction when compared to Ctrl or Ad-Ctrl treated cells (**Figures 1D–F**). We also performed western blotting on whole cell lysates extracted from cells treated with Ctrl, Ad-Ctrl, 100U recombinant muIFNα, and muAd-Ifnα to confirm induction of muIFNα signaling in MB49 cells. Western blot analysis confirmed increased expression of phospho-Akt, phospho-Stat1, Irf7, Cdk4, and reduced expression for Cdkn2a and Cyclin D1, whereas expression for total-Akt and total-Stat1 were comparable between treatments (**Figure 1G**).

### 3.2 Preclinical assessment of muAd-Ifnα in an intravesically-generated MB49 tumor model

Next, we tested the anti-tumor efficacy of intravesical muAd-Ifnα treatment in the syngeneic MB49 BLCA model and compared it with vehicle-treated or Ad-Ctrl treated mice. The study’s schematics are shown (**Figure 2A**). Intravesical administration of muAd-Ifnα significantly improved survival (p=0.0005) (47 days) when compared to Ad-Ctrl (34.5 days) or vehicle (31 days) (**Figure 2B**). Bladder weights in end-stage tumors showed decreased weights in muAd-Ifnα treated tumors (**Figure 3C**). Histological assessment of murine bladder tumors revealed reduced tumor burden and decreased proliferation (Ki67 immunohistochemistry) in muAd-Ifnα treated tumors when compared to Ad-Ctrl treated tumors (**Figure 2D**; panel H&E and panel Ki67). We performed immunohistochemistry for CD4 and CD8 immune cell markers to investigate the intratumoral immune cell composition of these two cell types specifically as these cell types were altered in our preclinical models (4, 21). CD4 T-cell numbers were decreased whereas CD8 T-cell numbers remained comparable between vehicle, Ad-Ctrl, and muAd-Ifnα treated cells (**Figure 2D**, panel CD4, and CD8), and quantification of the cell numbers also confirmed the same results (**Figures 2E, F**).

### 3.3 High throughput 10X scRNAseq analysis of intravesical MB49 tumor model

To test the efficacy of the muAd-Ifnα vector on the tumor microenvironment, we established an intravesical BLCA model by intravesical instillation of luciferase-tagged MB49 cells into the bladder of female mice (8 weeks old). Tumor establishment was verified by IVIS imaging after 1 week. Mice with established tumors were randomized and treated with either Ad-Ctrl or muAd-Ifnα vectors by intravesical instillation. Single cell suspensions from treated tumors were prepared as discussed in the methods section. A schematic representation of the experimental protocol is shown (**Figure 3A**). Quality control parameters, mitochondrial gene percentages) and number of RNA features were assessed for each of the samples to filter low quality cells (**Supplementary Figure 1B; a, b**). We identified 16 distinct cell clusters in Ad-Ctrl and muAd-Ifnα treated samples (**Supplementary Figure 1B; c**). Clusters 8, 10, 14, and 15 were not included in the further analysis as they contained fewer transcripts as shown in the violin plots (**Supplementary Figure 1B; d**). The rest of the cell clusters were included for subsequent analysis. Based on marker expression, clusters 0, 1, 2, 3, 4, 6, 7, and 9 were grouped as epithelial, cluster 12 as uroplakin-enriched, cluster 13 as endothelial cells, cluster 11 as T-cell cluster, cluster 5 as macrophages, and cluster 10 as neutrophils (**Figure 3B**). These clusters were present in both Ad-Ctrl and muAd-Ifnα treated samples (**Figures 3C, D**). Expression of markers representing different clusters are shown, including endothelial marker (Vcam1), epithelial markers (Krt8, Krt18, Krt19, Krt5), uroplakins (Upk1a, Upk1b, Upk2, Upk3a), T-cell markers (Cd3d, Cd3g, Cd3e), macrophage markers (Adgre1, Csf1r, Mafb, Cd14, Fcgr3) and neutrophil markers (Csf3r, S100a8, S100a9, Cxcl3) (**Figure 3E**).

### 3.4 Comparison of genes/pathways between Ad-Ctrl and muAd-Ifnα in the tumor cell cluster

Implanted tumor cells are the main epithelial cell clusters that we identified in this study. To identify differences in gene expression between Ad-Ctrl and muAd-Ifnα, we performed differential gene expression between the two groups and identified 328 differentially expressed genes (DEGs) (FDR 0.05). A volcano plot representing the top differentially regulated genes including upregulated genes (Ifi2712a, Irf7, Slfn8, Oas2, Oas1g, Oas1a, Trim30d, Dhx58, and Csprs) and downregulated genes (Cd74, C1qb, Serpina3, Vcam, Fst, Gstt1, etc.) is shown (**Figure 4A**). A complete list of differentially expressed genes is shown in **Supplementary Table 1**. Next, we compared the gene expression between Ad-Ctrl and muAd-Ifnα treated groups to identify differentially regulated pathways using IPA. In this cluster, IFNα signaling (z-score: 2.121) and hypercytokinemia/chemokinemia (z-score: 4.243) were upregulated (**Figure 4B**). Commonly upregulated cell-death related pathways included death receptor signaling pathway (z-score: 2.449), necroptosis pathway (z-score: 2.333), and RA-mediated apoptotic signaling pathway (z-score: 2). A complete list of differentially expressed pathways is shown in **Supplementary Table 2**. Next, we analyzed changes in the gene expression of significantly altered genes in this cluster at the single-cell level. IFNα signaling molecules Stat2, Irf9, and Tlr3 were significantly upregulated in the muAd-Ifnα treated cells at the single-cell level, as shown in the violin plots (**Figures 4C–E**). Cytokines Ccl7 and IL33 were upregulated in the muAd-Ifnα treated cells (**Figures 4F, G**). Daxx, Tnfsf10, and Parp11, which are cell death pathway genes, were also significantly upregulated in the treated cells (**Figures 4H–J**).

Similar analysis of the uroplakin-enriched cell cluster showed 109 DEGs including upregulated (Ifi2712a, Plac8, Ifitm3, Bst2, etc.) and downregulated genes (H2-Aa, H2-Ab1, Fam3b, Ido1, Depp1, Ifi27, etc.) (**Figure 5A**). A complete list of genes is shown in **Supplementary Table 3**. In addition, the uroplakin-enriched cluster showed positive enrichment for hypercytokinemia/chemokinemia (z-score: 4.123), IFNα signaling (z-score 2.887), death receptor signaling (z-score: 2.646), RA-mediated apoptotic pathway (z-score: 1.633), necroptosis pathway (z-score: 2.887), wound healing signaling pathway (z-score: 3.051), pathogen-induced cytokine storm signaling pathway (z-score: 1.961) and activation of IRF by cytosolic pattern recognition receptor molecules (z-score: 1.508) (**Figure 5B**). A complete list of significant pathways is shown in **Supplementary Table 4**. Next, we compared gene expression between Ad-Ctrl and muAd-Ifnα at the single-cell level. IFNα signaling genes (Stat1, Stat2, Zbp1, Eif2ak2), cell death pathway genes (Tnfsf10, Parp9, and Parp14), and chemokines (Cxcl10) were all upregulated in muAd-Ifnα treated cells when compared to Ad-Ctrl treated cells (**Figures 5C–J**). However, Tnfsf10, one of the effector genes involved in multiple cell death pathways, was only slightly upregulated (Exp log ratio 0.03) in comparison to Ad-Ctrl treated cells.

### 3.5 Comparison of genes/pathways in endothelial cells

To identify changes in endothelial cell clusters, we performed differential expression analysis between the Ad-Ctrl and muAd-Ifnα treated cells. A total of 296 genes were differentially expressed (FDR 0.05, **Supplementary Table 5**). A volcano plot showing differentially expressed upregulated genes (Apod, Ifit3, Rsad2, Isg15, Ifi44, Ifit1, Irf7, Usp18, Ifit3b, and Phf11d) and downregulated genes (Ubd, Acta2, Actg2, Ramp3, Cxcl9, Myl9, Higd1b, Tagln, IL1b, and Tmsb4x) is shown (**Figure 6A**). We also identified differentially expressed pathways: IL-10 signaling (z-score: 2.746), IFN signaling (z-score: 2.309), role of hypercytokinemia/chemokinemia (z-score: 3.3), necroptosis pathway (z-score: 3) were upregulated whereas EIF2 signaling (z-score: -4.416), ILK signaling (z-score: -3.3), integrin signaling (z-score: -3.873) and actin cytoskeleton signaling pathway (z-score: -3.3) were downregulated in treated cells (**Figure 6B**). A complete list of pathways is shown in **Supplementary Table 6**. We also analyzed changes at the single cell level and found IFN signaling pathway genes Stat1, Stat2, and Tlr3 were upregulated in treated cells (**Figures 6C–E**), death receptor molecule Tnfsf10 and actin cytoskeletal genes Actb and Actg1 were downregulated in treated cells (**Figures 6F–H**). Cytokines IL1β and its receptor IL1r2 were also downregulated (**Figures 6I, J**). We also directly tested the effect of muAd-Ifnα on murine endothelial cell line 2H-11. 2H-11 cells could be transduced with muAd-Ifnα vector *in vitro* and expressed muAd-Ifnα protein (795.8 pg/ml) at 72h post-transduction (**Figure 6K**), decreased cell numbers after 72h (**Figure 6L**), and increased apoptotic cell numbers following treatment (**Figure 6M**). We also performed a tube formation assay, a standard assay that quantifies the ability of endothelial cells to form tubes in the presence of compounds that promote or inhibit tube formation (22). Treatment with type I IFN decreased angiogenesis in our previous studies (4, 23). In the 2h-11 cells treated with Ad-IFNα, tube formation was significantly reduced when compared to control cells (**Figure 6N**).

### 3.6 Comparison of genes/pathways in immune cells of the tumor microenvironment

We previously showed that IFNα treatment engaged both innate and adaptive immune cells in an immunocompetent subcutaneous mouse model using syngeneic MB49 cells (4). T-cells are the main effector cells of adaptive immunity that mediate anti-tumor responses (24), so we first focused on these cells. To identify changes induced by gene therapy, we performed differential gene expression (FDR 0.05) and identified 106 genes to be differentially regulated. A volcano plot with significantly upregulated genes (Spp1, Ly6c2, Usp18, Isg15, Mcpt8, Irf7, Ifit3, Bst2, Ifi2712a, Gzmf, etc.) and downregulated genes (Cxcl9, Cd74, H2-Eb1, H2-Aa, Lyz2, IL1b, H2-Ab1, Trac, and Tnfrsf4) is shown (**Figure 7A**). A complete gene list is provided in **Supplementary Table 7**. We also performed IPA analysis on the DEGs and found significant positive enrichment for IFNα signaling (z-score: 2.53), role of hypercytokinemia/chemokinemia (z-score: 2.357), immunogenic cell death signaling pathway (z-score: 2.496) and negative enrichment for Th2 pathway (z-score: -1.807) (**Figure 7B**). A complete list of pathways is provided (**Supplementary Table 8**). We analyzed gene expression of IFNα target genes Stat1 and Irf7 in this cell cluster and both genes were upregulated significantly in muAd-Ifnα treated cells (**Figures 7C, D**). Granzymes are a class of proteins that are markers for activated T-cells with cytotoxic functions (25). We found significant upregulation of Gzmb and Gzmc in the T-cell cluster (**Figures 7E, F**). We identified several genes such as H2-Aa, H2-Ab1, A2-Eb1, and Tnfrsf4 genes that were downregulated after treatment (**Figures 7G–J**).

Next, we focused on the major cell cluster, macrophages, which is a part of the innate immune system that plays an important role in cancer therapy (26, 27) Differential expression of cells identified 264 genes to be significantly altered (FDR 0.05). A volcano plot with significantly upregulated (Ifit1bl1, Ifit3b, Ifit3, Usp18, Rsad2, Phf11d, Ifit1, Oasl1, Cmpk2, and Oas3) and downregulated genes (Hbb-bs, Cxcl9, Ccl24, H2-Aa, H2-Ab1, Cd74, Ciita, Hba-a1, H2-Eb1, and Ifitm1) is shown (**Figure 8A**). A complete gene list of the differentially expressed genes are shown in **Supplementary Table 9**). We also performed IPA analysis on the differentially expressed genes (**Figure 8B**). Hypercytokinemia/chemokinemia (z-score: 1.964), IFNα signaling pathway (z-score: 1.414), MSP-RON signaling pathway (z-score: 2.496) and PD1, PD-L1 pathway (z-score: 1.897) were upregulated in Ad-IFNα treated cells. Macrophage classical activation pathway (z-score: -2.043), macrophage alternate activation pathway (z-score: -2.414), Th1 pathway (z-score: -1.604), and Th2 pathway (z-score: -1.5) were downregulated in this cell cluster after treatment. A complete list of pathways is provided (**Supplementary Table 10**).

We also compared the relative expression of IFNα target genes, Stat2 and Irf7, at the single-cell level. Expression of the IFNα target genes were significantly upregulated in the treated cluster (**Figures 8C, D**). We looked for the expression of cytokines involved in the classical activation of macrophages. Ccl5, IL1β, and Hif1α were downregulated in this cluster after treatment (**Figures 8F, G**). MSP-RON signaling is an important pathway that plays a role in tissue microenvironment (27). This pathway was significantly upregulated and genes Ciita, H2-Dma, H2-Dmb1, H2-Aa, H2-Ab1, H2-Eb1, and Ptgs2 were downregulated after treatment (**Figures 8H–N**).

### 3.7 Multiplex immunofluorescence analysis comparing control with muAd-Ifnα treated cells

**Figure 9:**
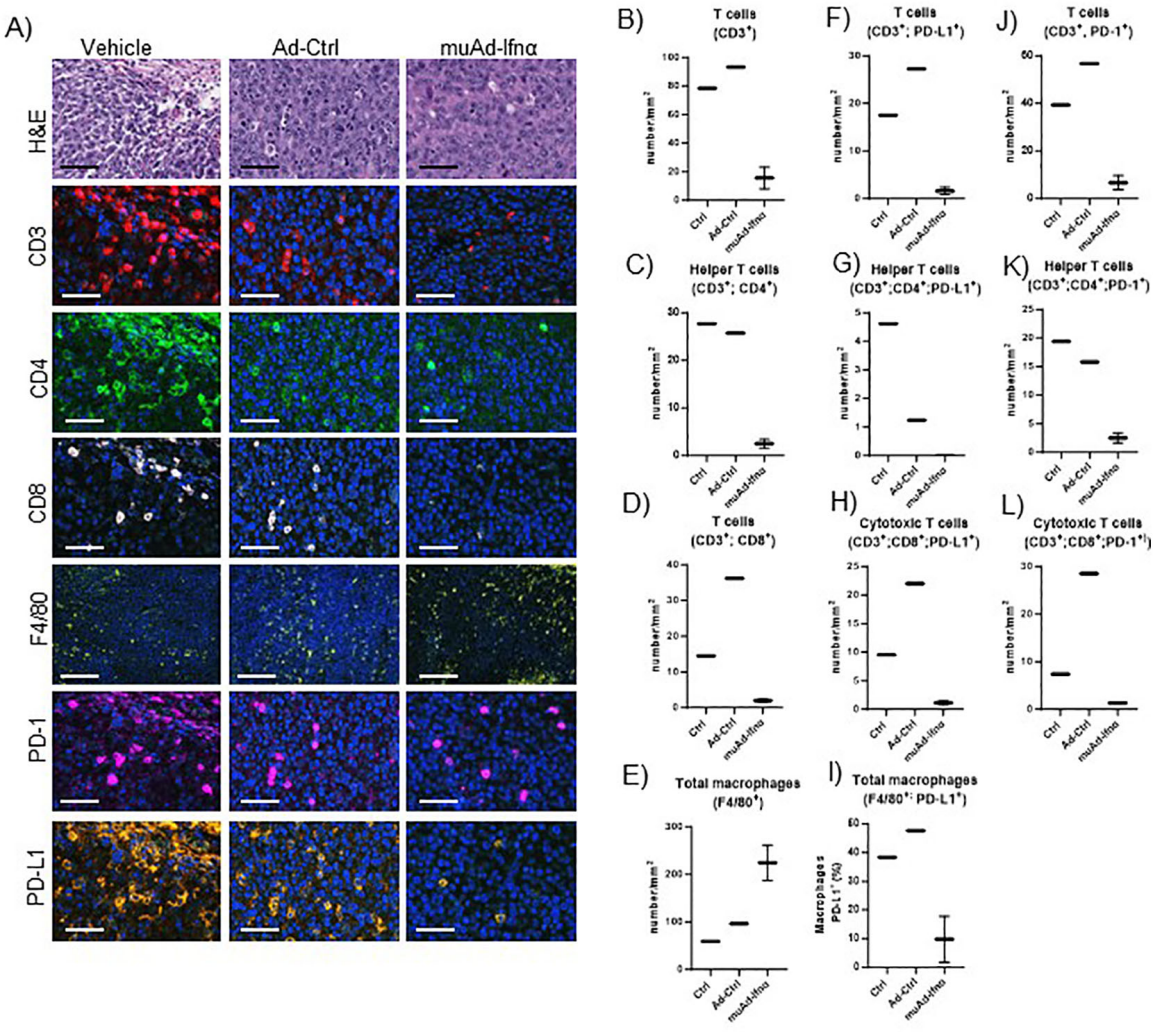
Multiplex immunofluorescence of MB49 tumors 72h post-treatment. Microscopic images of tumors H&E, CD3, CD4, CD8, F4/80, PD-1, and PD-L1 comparing vehicle with Ad-Ctrl and muAd-Ifnα treated tumors **(A)**. Quantification of total T cells (CD3+, **B**), helper T cells (CD3+; CD4+, **C**), cytotoxic T cells (CD3+; CD8+, **D**) and macrophages (F4/80+, **E**). Quantification of PD-L1 in total T cells (CD3+; PD-L1+, **F**), helper T cells (CD3+; CD4+; PD-L1+; **G**), cytotoxic T cells (CD3+; CD8+; PD-L1+, **H**), and macrophages (F4/80+; PD-L1+, **I**). Quantification of PD1+ in total T cells (CD3+; PD-1+, **J**), helper T cells (CD3+; CD4+; PD-1+, **K**), and cytotoxic T cells (CD3+; Cd8+; PD-1+, **L**).

To validate our scRNA sequencing data, we performed multiplex immunofluorescence using a murine panel as described in the materials and methods section. Representative images of the cell types expressing general T-cells (CD3^+^), helper T-cells (CD3^+^, CD4^+^), cytotoxic T-cells (CD3^+^, CD8^+^), and macrophages (F4/80+) are shown in **Figure 9A**. Quantification of these cells showed a reduction in cell numbers of CD3, CD4, and CD8 T-cells, while macrophages were increased (**Figures 9B–E**). MSP-RON signaling is thought to upregulate checkpoint molecules, and IFNα signaling has been shown to upregulate PD-L1 expression (25). We checked for expression of PD-1 and PD-L1 by immunofluorescence in the immune cell compartments. A representative image is shown in **Figures 9A**. Quantification of total PD-L1^+^ cells in CD3+ cells, helper T-cells, cytotoxic T-cells, and macrophages showed reduced cell numbers (**Figures 9F–I**). A similar quantification showed reduced PD-1 expression in CD3+ cells, helper T-cells, and cytotoxic T-cells (**Figures 9J–L**). While untreated control tumors and Ad-Ctrl tumors showed a similar number of immune subsets, there were some exceptions. For example, PD-L1 positive helper T cells were significantly lower in the Ad-Ctrl treated cells when compared to control samples. CD8+ (PD-L1+ and PD-1 +) cells were also highest in the Ad-Ctrl group. We believe this is due to the immune response to the adenoviral vectors, which we are currently investigating.

We performed a similar analysis on the neutrophil cell cluster, and only 33 genes were differentially expressed in the neutrophil cluster. The volcano plot with DEGs is shown in **Supplementary Figure 2A**. A complete list of genes is provided in **Supplementary Table 11**. IPA identified IFNα signaling, pathogen-induced cytokine storm, and hypercytokinemia/chemokinemia as the main pathways upregulated in muAd-Ifnα (**Supplementary Figure 2B**). A complete list of pathways is shown in **Supplementary Table 12**.

## 4 Discussion

With the recent FDA approval, Nadofaragene firadenovec has emerged as a breakthrough therapy for patients with BCG-unresponsive NMIBC and represents a promising second-line option for patients who elect bladder-sparing treatment or are ineligible for cystectomy (2). IFNα is a pleiotropic cytokine, and its complex antitumor effects involve multiple cell types in the TME (28, 29). In this study, we report the development of a murine adenoviral vector expressing IFNα protein (muAd-Ifnα) and test its efficacy in murine BLCA cell lines and animal models. We confirmed the successful transduction of viral particles into BLCA cells with significant production of IFNα protein in cell-free supernatants and demonstrated the induction of cell death and IFN target genes at both the RNA and protein levels. Intravesical instillation of these vectors in syngeneic MB49 models significantly improved survival in mice, consistent with what we observed with the lentiviral vectors (LV-IFNα) (21). The adenoviral vectors are currently approved by the FDA for use in humans with bladder cancer (30). Analysis of these vectors in murine models is highly relevant and important for understanding treatment responses observed in patients.

To better understand the dynamics of Ad-IFNα gene therapy, we generated scRNAseq data of bladders treated with muAd-Ifnα vectors and compared that with the Ad-Ctrl treated cells at 72h post-treatment. We identified 16 distinct clusters; based on literature (31, 32) and marker expression we were able to assign these clusters into epithelial (MB49 tumor cluster, Krt8^+^, Krt18^+^), uroplakin-enriched cluster representing the normal urothelium of the bladder (Krt8^+^, Krt18^+^, Krt19^+^, Krt5^+^, Upk1a^+^, Upk1b^+^, Upk2^+^ and Upk3a^+^), endothelial cluster (Vcam^+^), T-cell cluster (Cd3d^+^, Cd3g^+^, Cd3e^+^), macrophages (Adgre1^+^, Csf1r^+^, Mafb^+^, Cd14^+^, Fcgr^+^), and neutrophils (Csf3r^+^, S100a9^+^, S100a9^+^, Cxcl3^+^). Consistent with published data, MB49 cells lacked expression of urothelial keratins Krt5, Krt14, and uroplakins (33) and were identified by expression of Krt8 and Krt18.

IFNα signaling and expression of several cytokines/chemokines were upregulated and resulted in upregulation of several cell death signaling pathways including death receptor signaling, retinoic acid cell signaling, and necroptosis signaling. Cell death induced by tumor necrosis factor-related apoptosis-inducing ligand (TRAIL, Tnfsf10) has been shown to contribute to anti-tumor effects in epithelial cells including BLCA cells (8, 34) (8, 31). Consistent with our previous studies, TRAIL was upregulated in this cluster (Exp Log Ratio: 1.709) and possibly contributes to cell death of epithelial cells *in vivo*. In contrast to this, TRAIL expression was only very slightly upregulated in the uroplakin-enriched normal urothelial cells (Exp Log Ratio, 0.03), consistent with the role of TRAIL-induced apoptosis restricted to malignant cells and sparing normal cells such as the urothelial cells (5, 35). DAXX protein is involved in the interferon-triggered pathway of apoptosis where it is translocated to the nucleus and suppresses cell-cycle related genes (36); Daxx was also upregulated in the treated cells.

IFNα also limits tumor growth via its anti-angiogenic effects that involve several mechanisms such as inhibition of proangiogenic factors such as basic FGF, IL-8, and VEGF (37, 38). IFNα protein can also directly impair the proliferation and migration of endothelial cells *in vitro* (39). In this study, we found significant downregulation of IL-1β along with its receptor IL-1r2 in the endothelial cells. Tumor microenvironment-derived IL-1β is an important mediator for angiogenesis and inhibition of IL-1 and IL-1 receptor antagonists reduced angiogenic response and tumor growth (40, 41). Other pro-angiogenic factors PDGFα and PDGFβ which are important for vessel formation and stability (42) were also significantly downregulated in treated cells. In addition to this, actin cytoskeletal signaling, EIF2 signaling, integrin, and ILK signaling were all downregulated in treated endothelial cells. Actin cytoskeletal organization is important for blood vessel formation and in regulating endothelial cell function (43). In our study, significant downregulation of the pathways that are necessary for blood vessel integrity was noted. Lastly, TRAIL expression was also significantly upregulated in the endothelial cells suggesting cell death induced by muAd-Ifnα. We further validated these findings by direct transduction of murine endothelial cell line 2H-11 with muAd-Ifnα vectors and showed successful transduction with protein induction, cell death, defective vessel formation, and actin cytoskeletal changes.

T cells are the main effectors of adaptive immunity mediating antitumor responses, and IFNα protein increases the activation of T lymphocytes (44). Markers Cd3d, Cd3e, and Cd3g identified cells in the T cell cluster. As expected, IFNα signaling mediated by Stat1, Irf7, and other ISGs were upregulated in treated cells. Cytotoxic T cells express granzymes such as Gzmc when activated by IFNα proteins (45, 46), suggesting that the treated cells were responding to IFNα.

Macrophages represent the largest cell cluster in our analysis, and tumors showed increased infiltration of macrophages. In this cluster, IFNα signaling and hypercytokinemia/chemokinemia were upregulated along with MSP-RON signaling. In the macrophage cluster, cytokines Ccl22, Ccl5, and I1b, which are known to have protumor activity were all downregulated. This has a positive impact on the antitumor response. Also, Ccl2, Ccl7, and IL-15 which have anti-tumor responses were upregulated in this cluster, which likely contributes to the IFNα-mediated antiphagocytic activity of macrophages (47, 48).

Increased recruitment of macrophages was validated using multiplex immunofluorescence. PD-1/PD-L1 expression was downregulated after treatment at 72h timepoint in responsive tumors. To understand the clinical relevance of this finding, we also assessed PD-1 and PD-L1 expression in pre-treatment and post-treatment phase 3 clinical samples, and significant upregulation of expression was found in non-responders at 3 months and the final outcome suggesting an acquired resistance mechanism that can be targeted using PD1 or PD-L1 inhibitors (49).

There are some limitations of this study. We only assessed the 72h time point, which limits our ability to describe temporal changes induced by muAd-Ifnα and warrants further investigation. Moreover, we used an intravesical MB49 model, which is not a perfect model to represent NMIBC. Additional studies using genetically engineered mice (e.g. carcinogen-induced mouse models (50) and luminal cancer models (51) after acute and chronic exposure to gene therapy will be critical for clarifying innate and adaptive responses and in identifying clinically actionable combination strategies.

**Figure 10:**
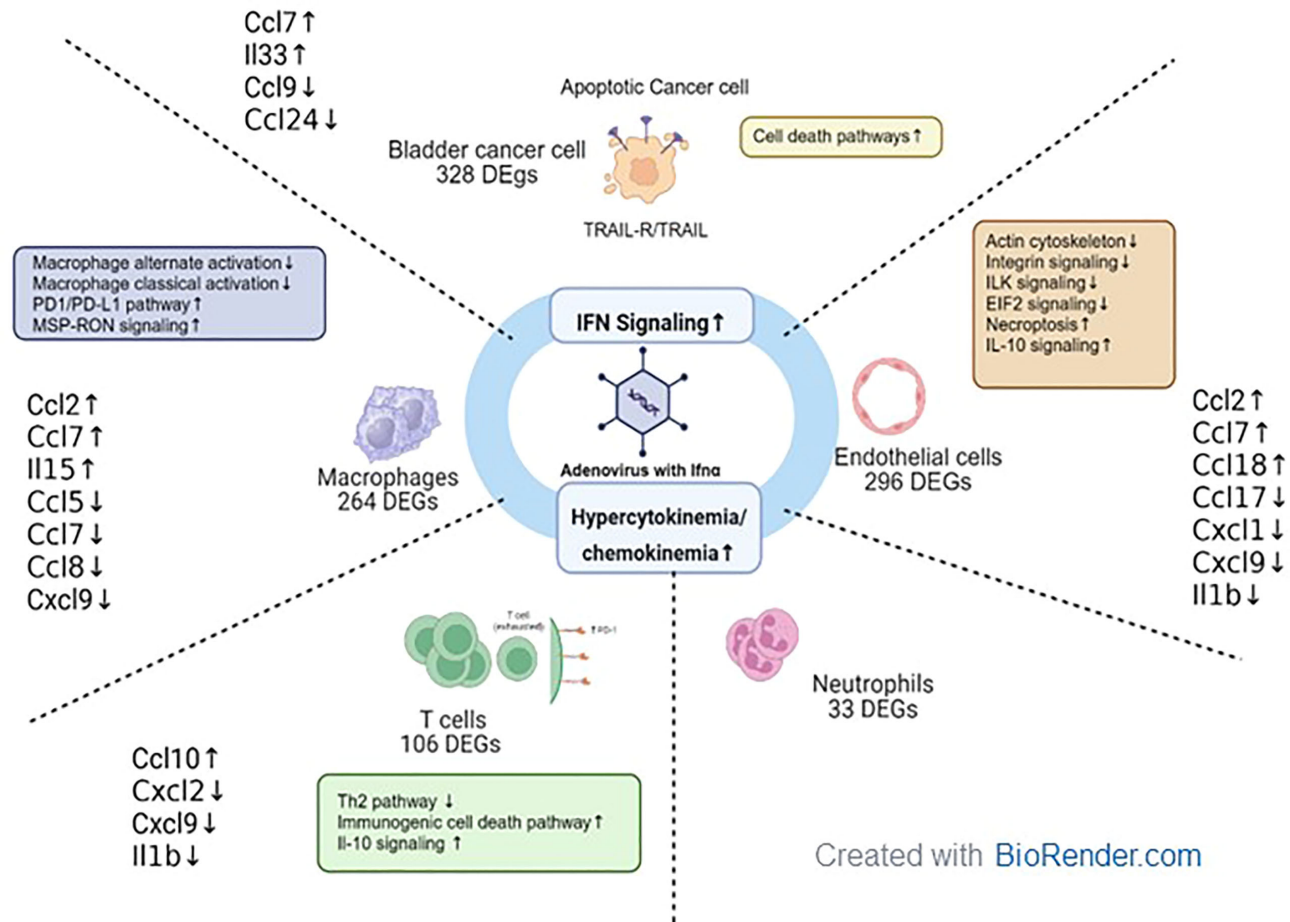
Tumor microenvironment changes in mice treated with muAd-Ifnα gene therapy. muAd-Ifnα expressing adenoviral particles were developed to treat murine BLCA models. Short-term exposure of murine tumors treated with gene therapy vectors resulted in significant changes in cell clusters of the tumor microenvironment and DEGs altered in each cell type are shown. All cell types showed significant upregulation of IFNα signaling and hypercytokinemia and chemokinemia pathways. Uniquely epithelial cells showed upregulation of cell death pathways, while T cells showed upregulation of immunogenic cell death pathways, IL-10 signaling, and downregulation of the Th2 pathway. Macrophages showed upregulation of the Pd1/PD-L1 pathway and MSP-RON pathway while classical and alternate activation pathways of macrophages were downregulated. Endothelial cells were characterized by downregulation of actin cytoskeletal signaling, ILK signaling, integrin signaling, and increased necroptosis and IL-10 signaling.

In summary, we developed adenoviral vectors expressing muIFNα for the first time and tested these vectors in cells and murine intravesical BLCA model using MB49. Using scRNAseq on tumors treated with muAd-Ifnα vectors, we were able to demonstrate IFNα pathway activation in all cell clusters, and in the tumor cells antitumor properties were induced by activation of apoptotic pathways, while in the endothelial cell apoptosis, actin remodeling and inhibition of IL-1b and lL-1r2 contributed to anti-angiogenic effect. We observed activation of T-cells and infiltration of macrophages with decreased classical and alternate activation pathways but with cytokine expression favorable for anti-tumor responses. Our results are summarized in **Figure 10**.

## Data Availability

The original contributions presented in the study are included in the article/**Supplementary Material**, further inquiries can be directed to the corresponding author.

## Supplementary material

Supplementary Figure 1
**(A)** Western blot analysis for muIfnα expression in cell lines transfected with vectors. **(B)** Quality parameters for scRNA sequencing analysis. Percent mitochondrial genes in the samples tested (a). Distribution of the number of transcripts per cell in each sample (b). Identification of cell clusters with tSNE plot showing 16 cell clusters (c). mRNA feature in the various clusters. Clusters 8, 10, 14, and 15 (marked by arrows) have very few transcripts and were eliminated from further analysis (d).

Supplementary Figure 2 scRNA seq analysis of neutrophil cell cluster. Volcano plot showing DEGs up and downregulated in the neutrophils (FDR 0.05; log2 FC1) **(A)**. IPA showing differentially regulated pathways in neutrophil cell cluster treated with muAd-Ifnα **(B)**.

Supplementary Table 1 Complete list of differentially expressed genes between Ad-Ctrl and muAd-Ifnα in the epithelial (tumor) cell cluster.

Supplementary Table 2 Complete list of differentially expressed pathways between Ad-Ctrl and muAd-Ifnα in the epithelial (tumor) cell cluster.

Supplementary Table 3 Complete list of differentially expressed genes between Ad-Ctrl and muAd-Ifnα in the uroplakin-enriched cell cluster.

Supplementary Table 4 Complete list of differentially expressed pathways between Ad-Ctrl and muAd-Ifnα in the uroplakin-enriched cell cluster.

Supplementary Table 5 Complete list of differentially expressed genes between Ad-Ctrl and muAd-Ifnα in the endothelial cell cluster.

Supplementary Table 6 Complete list of differentially expressed pathways between Ad-Ctrl and muAd-Ifnα in the endothelial cell cluster.

Supplementary Table 7 Complete list of differentially expressed genes between Ad-Ctrl and muAd-Ifnα in the T cell cluster.

Supplementary Table 8 Complete list of differentially expressed pathways between Ad-Ctrl and muAd-Ifnα in the T cell cluster.

Supplementary Table 9 Complete list of differentially expressed genes between Ad-Ctrl and muAd-Ifnα in the macrophage cluster.

Supplementary Table 10 Complete list of differentially expressed pathways between Ad-Ctrl and muAd-Ifnα in the macrophage cluster.

Supplementary Table 11 Complete list of differentially expressed genes between Ad-Ctrl and muAd-Ifnα in the neutrophil cluster.

Supplementary Table 12 Complete list of differentially expressed pathways between Ad-Ctrl and muAd-Ifnα in the neutrophil cluster.

Supplementary Methods Complete list of reagents used in the study.
