# Supplementary figures and images for: Single-cell RNA sequencing analysis identifies acute changes in the tumor microenvironment induced by interferon α gene therapy in a murine bladder cancer model
Source: Front Immunol. 2024 Nov 4;15:1387229. doi: 10.3389/fimmu.2024.1387229 (PMC11570268; doi:10.3389/fimmu.2024.1387229)

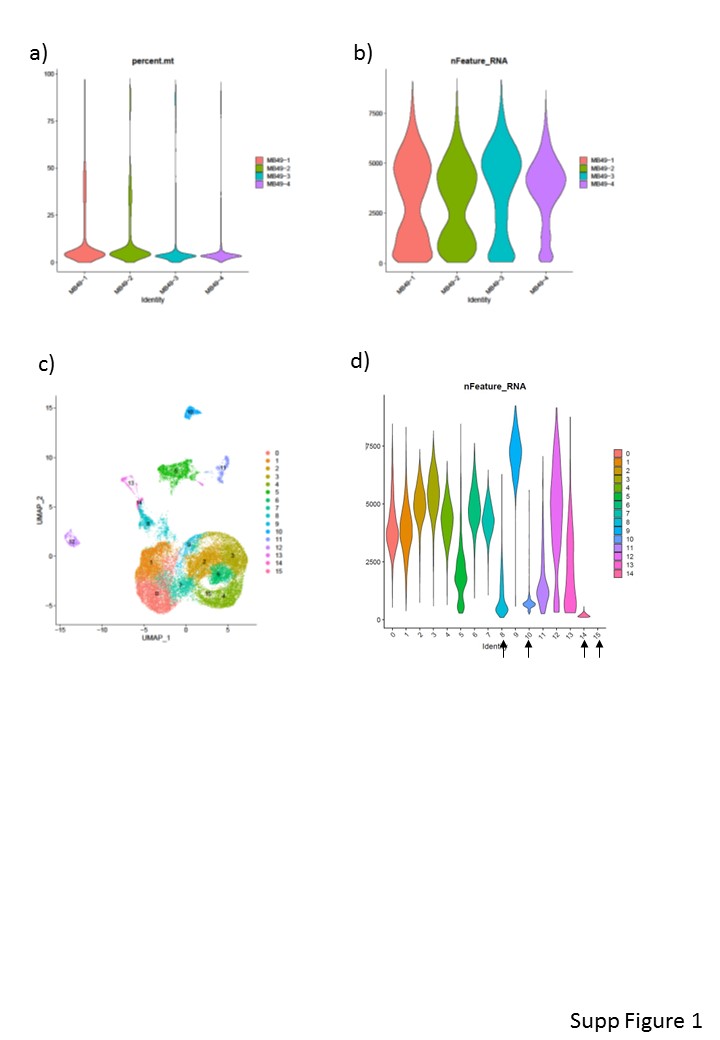

Supplement: Supplementary Figure 1 — (A) Western blot analysis for muIfnα expression in cell lines transfected with vectors. (B) Quality parameters for scRNA sequencing analysis. Percent mitochondrial genes in the samples tested (a). Distribution of the number of transcripts per cell in each sample (b). Identification of cell clusters with tSNE plot showing 16 cell clusters (c). mRNA feature in the various clusters. Clusters 8, 10, 14, and 15 (marked by arrows) have very few transcripts and were eliminated from further analysis (d). [file Image1.jpg]

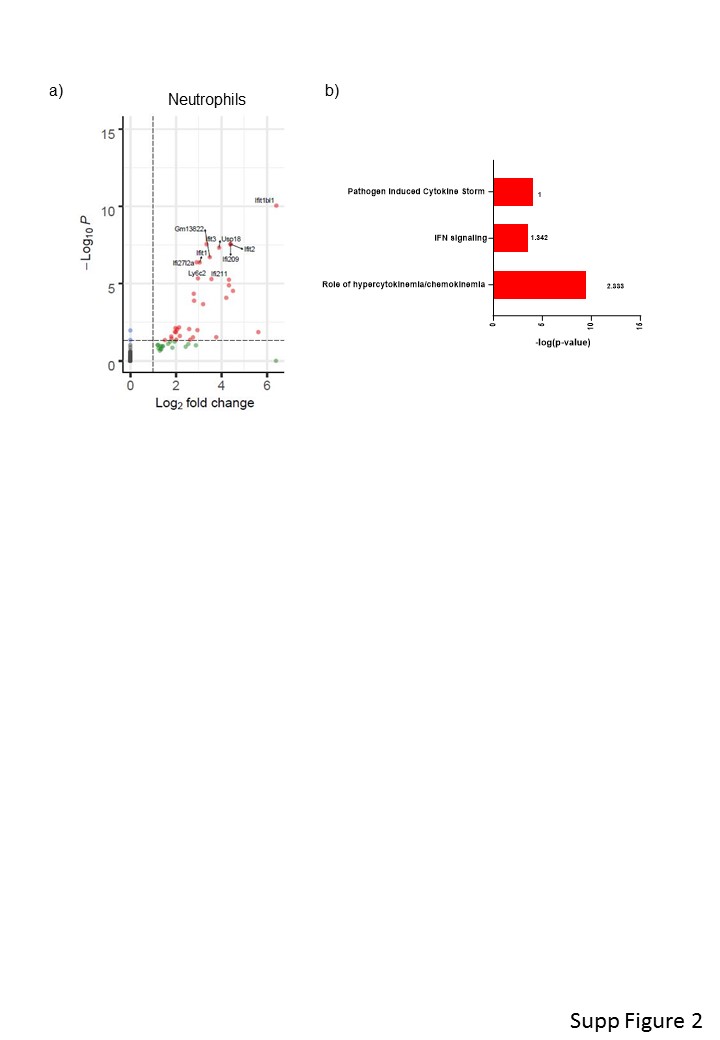

Supplement: Supplementary Figure 2 — scRNA seq analysis of neutrophil cell cluster. Volcano plot showing DEGs up and downregulated in the neutrophils (FDR 0.05; log2 FC1) (A). IPA showing differentially regulated pathways in neutrophil cell cluster treated with muAd-Ifnα (B). [file Image2.jpg]

## Slide 1
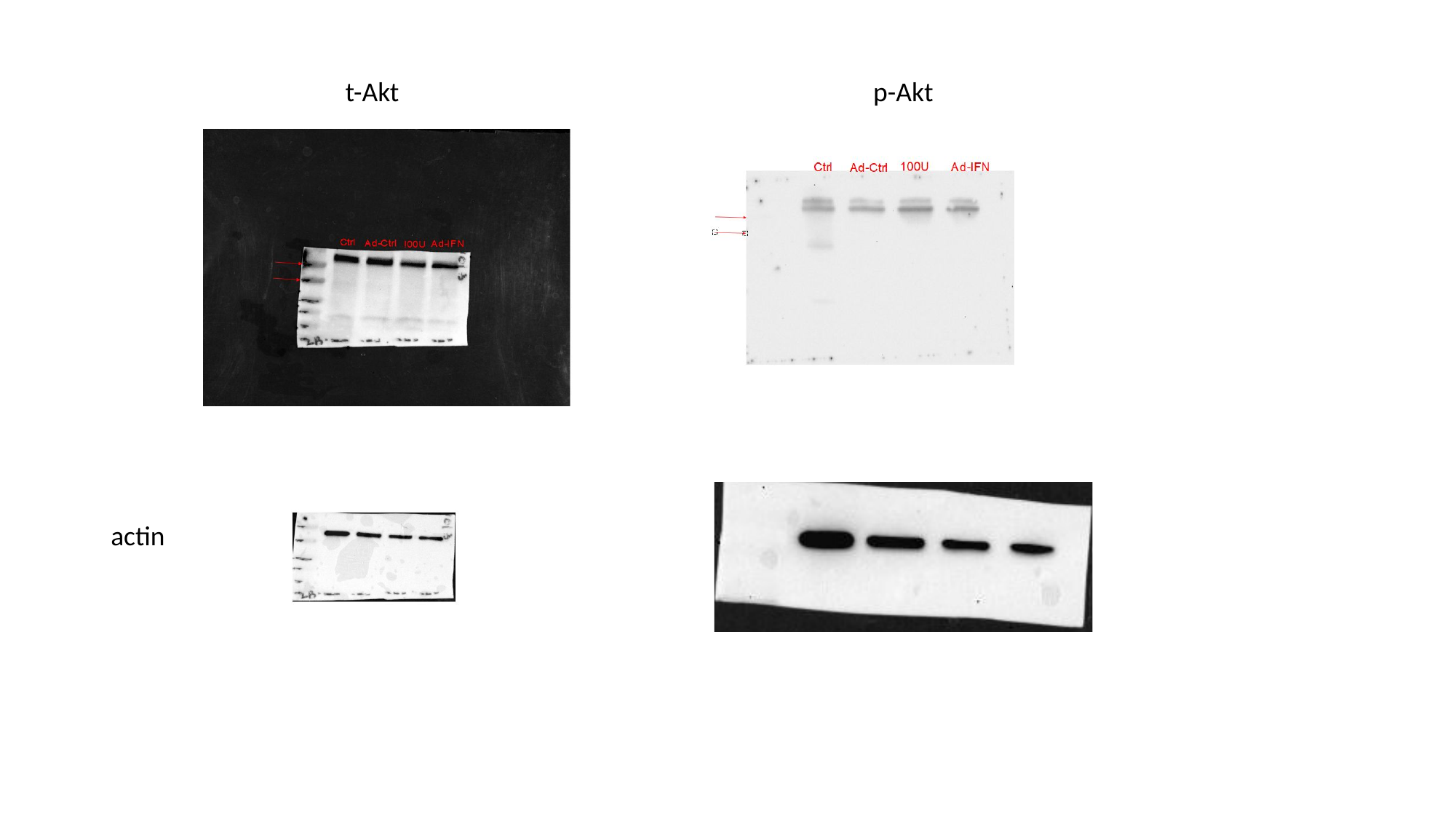

t-Akt
p-Akt
actin

## Slide 2
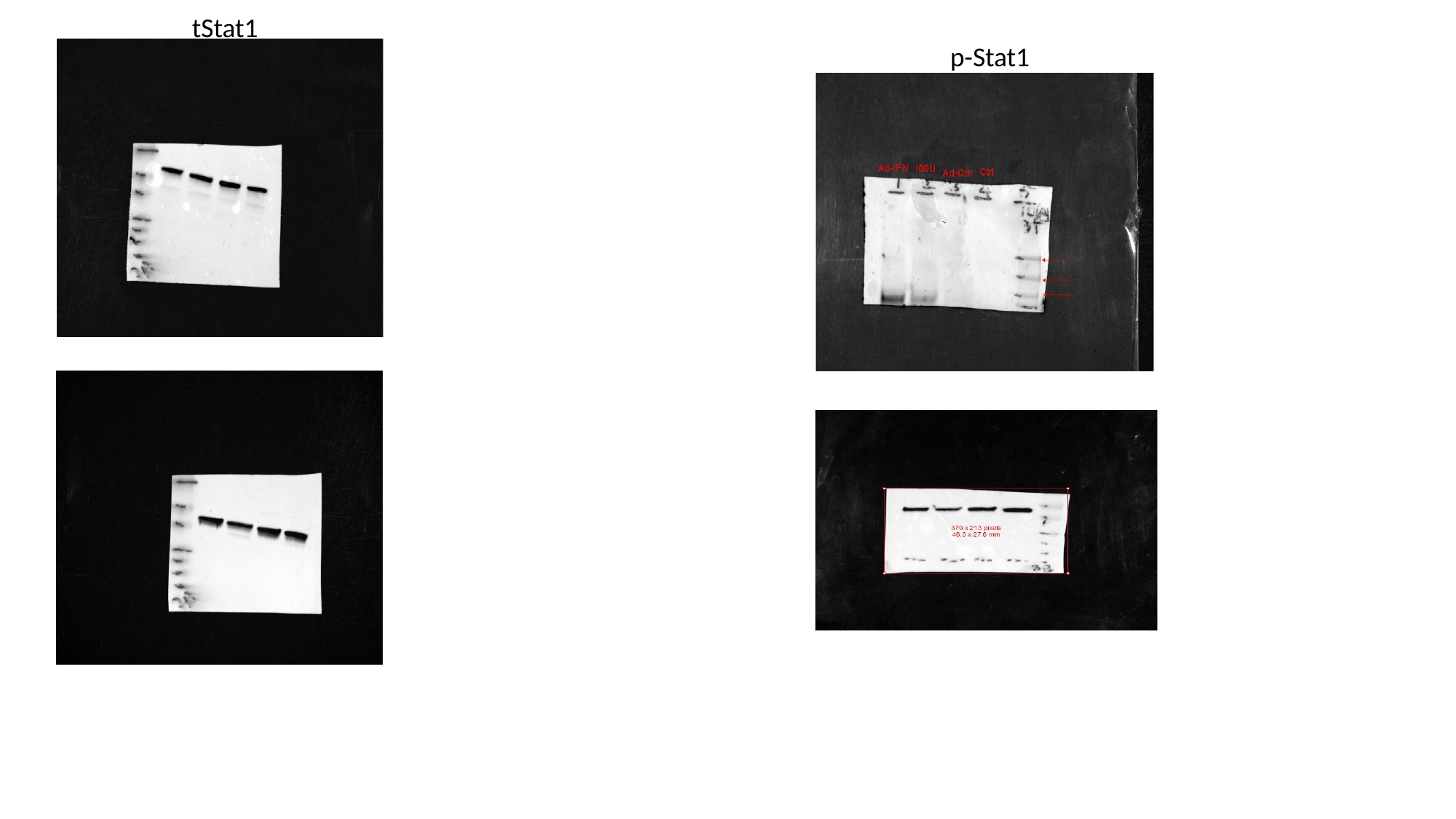

tStat1
p-Stat1

## Slide 3
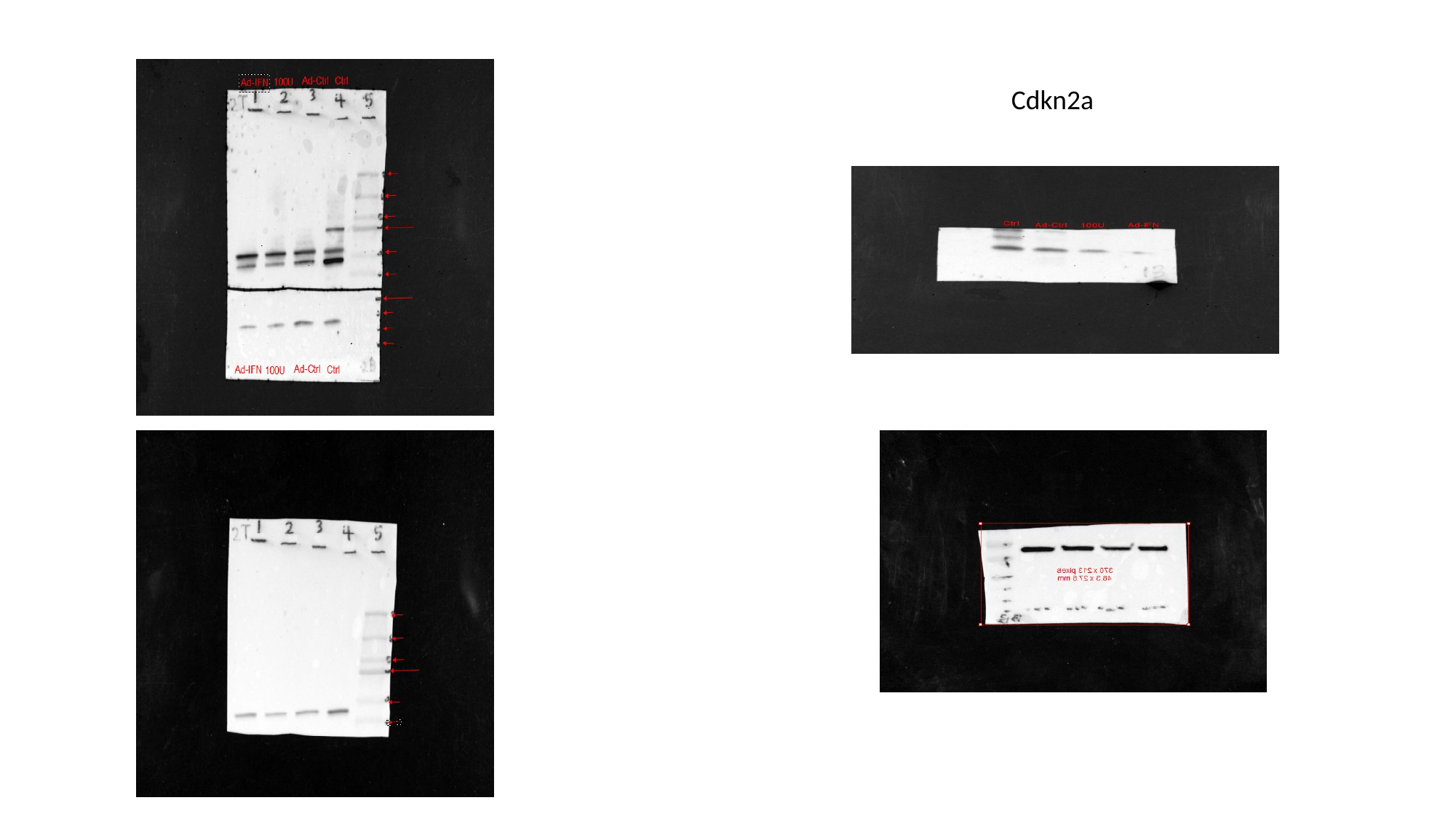

Irf7
Cdkn2a

## Slide 4
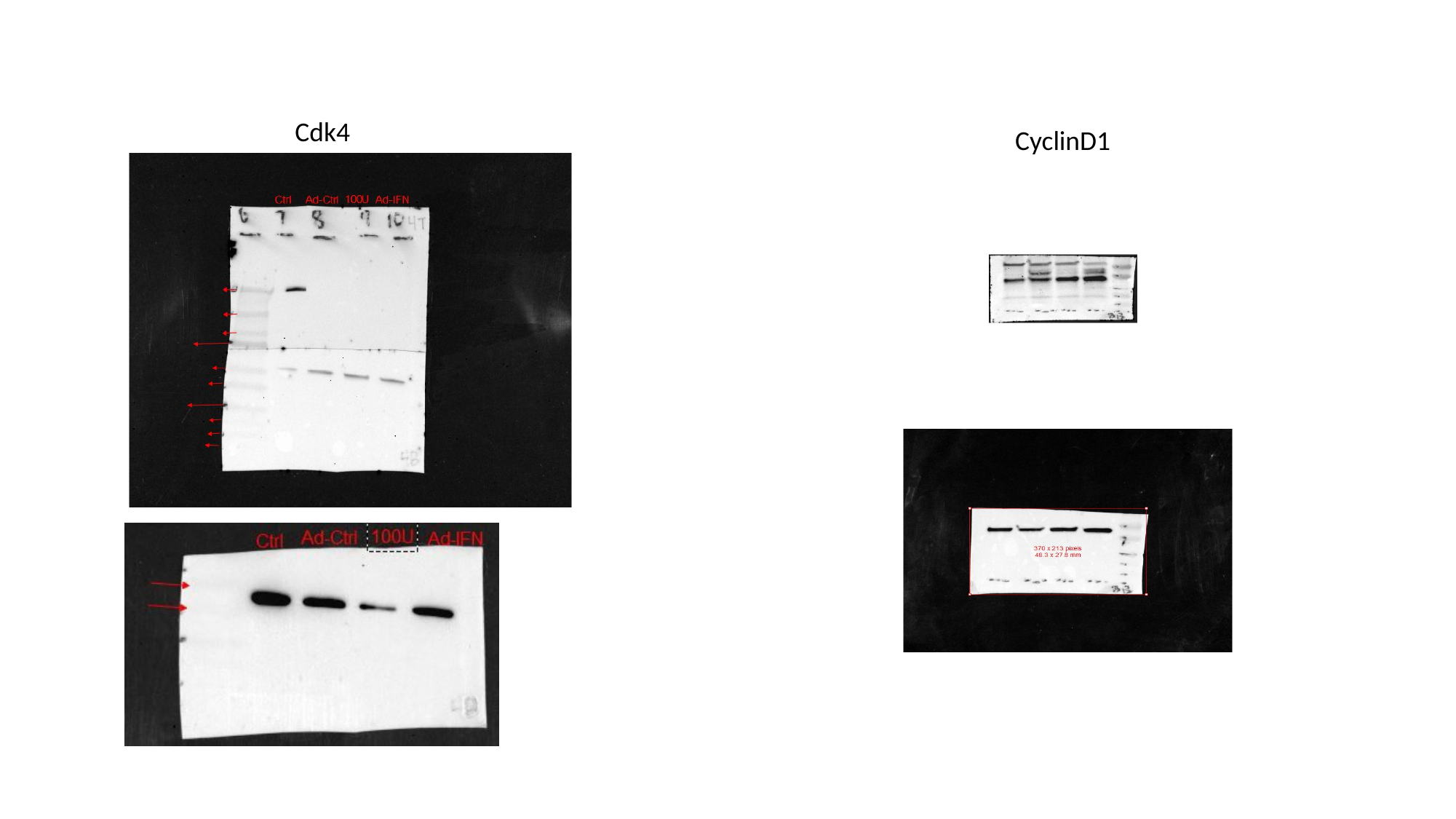

Cdk4
CyclinD1

Supplement: Supplementary file 22 [file Presentation1.pptx]
