# Supplementary material for: Single-cell RNA sequencing analysis identifies acute changes in the tumor microenvironment induced by interferon α gene therapy in a murine bladder cancer model
Source: Front Immunol. 2024 Nov 4;15:1387229. doi: 10.3389/fimmu.2024.1387229 (PMC11570268; doi:10.3389/fimmu.2024.1387229)
Supplement: Supplementary Methods — Complete list of reagents used in the study. [file DataSheet1.docx]

Supplementary methods

Source and concentration of reagents

| **S. No** | **Reagent** | **Source** | **Cat. #** |
| --- | --- | --- | --- |
| 1 | Minimum Essential media (MEM) | Corning | 10-010-CV |
| 2 | Penicillin/ Streptomycin | Corning | 30002 |
| 3 | Fetal Bovine Serum (FBS) | Sigma | F0926 |
| 4 | Polybrene | Sigma/Millipore | TR1003 |
| 5 | Recombinant murine IFNα | PBL Assay Science | 12100-1 |
| 6 | 2H-11 endothelial cell line | ATCC | CRL-2163 |
| 7 | Dulbeco’s modified Eagel’s medium (DMEM) | Corning | 10-017-CV |
| 8 | DPBS (10X) | Sigma | D1408 |
| 9 | Collagenase/Hylauronidase | Stem Cell Technologies | 7912 |
| 10 | Complete Mini Protease inhibitor cocktail | Roche | 11836153001 |
| 11 | PhosphoSTOP Phosphatase inhibitor cocktail | Roche | 04906845001 |
| 12 | Micro BCA protein assay kit | Thermo Scientific | 23235 |
| 13 | Permount | Fisher Chemical | SP15-100 |
| 14 | Trypsin (10X) 2.5% solution | Gibco | 15090-046 |
