# Supplementary material for: Single-cell RNA sequencing analysis identifies acute changes in the tumor microenvironment induced by interferon α gene therapy in a murine bladder cancer model
Source: Front Immunol. 2024 Nov 4;15:1387229. doi: 10.3389/fimmu.2024.1387229 (PMC11570268; doi:10.3389/fimmu.2024.1387229)
Supplement: Supplementary file 16 [file Table13.docx]

Data availability:

scRNAseq data: The data is uploaded and is available at the GEO database (GSE259337). It can be accessed with the following link and access token.

<https://www.ncbi.nlm.nih.gov/geo/query/acc.cgi?acc=GSE259337>

Access Token: **unuzkeuqhjmhzcd**
